# Can nanotechnology and genomics innovations trigger agricultural revolution and sustainable development?

**DOI:** 10.1007/s10142-024-01485-x

**Published:** 2024-11-16

**Authors:** Arzish Javaid, Sadaf Hameed, Lijie Li, Zhiyong Zhang, Baohong Zhang, Mehboob-ur -Rahman

**Affiliations:** 1 Plant Genomics and Molecular Breeding Laboratory, National Institute for Biotechnology and Genetic Engineering College, Pakistan Institute of Engineering and Applied Sciences (NIBGE- C, PIEAS), Faisalabad, 38000 Punjab Pakistan; 2 Faculty of Science and Technology, University of Central Punjab, Lahore, 54000 Pakistan; 3 School of Life Sciences, Henan Institute of Sciences and Technology, Xinxiang, 453003 Henan China; 4 Department of Biology, East Carolina University, Greenville, NC 27858 USA

**Keywords:** Nanobiotechnology, Nanoparticles, Genetic engineering, Genome editing, Sustainable development, Smart agriculture

## Abstract

At the dawn of new millennium, policy makers and researchers focused on sustainable agricultural growth, aiming for food security and enhanced food quality. Several emerging scientific innovations hold the promise to meet the future challenges. Nanotechnology presents a promising avenue to tackle the diverse challenges in agriculture. By leveraging nanomaterials, including nano fertilizers, pesticides, and sensors, it provides targeted delivery methods, enhancing efficacy in both crop production and protection. This integration of nanotechnology with agriculture introduces innovations like disease diagnostics, improved nutrient uptake in plants, and advanced delivery systems for agrochemicals. These precision-based approaches not only optimize resource utilization but also reduce environmental impact, aligning well with sustainability objectives. Concurrently, genetic innovations, including genome editing and advanced breeding techniques, enable the development of crops with improved yield, resilience, and nutritional content. The emergence of precision gene-editing technologies, exemplified by CRISPR/Cas9, can transform the realm of genetic modification and enabled precise manipulation of plant genomes while avoiding the incorporation of external DNAs. Integration of nanotechnology and genetic innovations in agriculture presents a transformative approach. Leveraging nanoparticles for targeted genetic modifications, nanosensors for early plant health monitoring, and precision nanomaterials for controlled delivery of inputs offers a sustainable pathway towards enhanced crop productivity, resource efficiency, and food safety throughout the agricultural lifecycle. This comprehensive review outlines the pivotal role of nanotechnology in precision agriculture, emphasizing soil health improvement, stress resilience against biotic and abiotic factors, environmental sustainability, and genetic engineering.

## Introduction

Over the last six decades, the Green Revolution remarkably tripled the global food production worldwide (The State of Food Security and Nutrition in the World 2019) which delayed the expected feminine in late 1970s. For providing sustainable food supply, to humans and animals, it is important to expand the crop productivity per unit area by 70% to meet the food demand of population beyond 2050 (Adisa et al. 2019). Notably, over 50–90% population of most developing countries residing in rural areas rely on agriculture to earn their livelihood (Munaweera et al. 2022).

Naturally, plants are surrounded by a set of complex environments making crops vulnerable to many stresses which have tremendous impact on crop production (Prakash et al. 2023; Sheri et al. 2023). Nanomaterials can be utilized as a strong weapon to lower environmental footprints thereby complementing crop husbandry and production (Khan et al. 2019b; Ur Rahim et al. 2021; Salama et al. 2021). Nano fertilizers and nano pesticides offer increased active ingredients and tunability in targeted delivery in crop production and protection (Rajput et al. 2021b; Chen et al. 2022; Zhi et al. 2022). Nano micronutrients enlighten innovations by imparting positive impacts on seed germination and crop improvement (Dulta et al. 2021). In short, nano inputs pave the way for possibilities of beneficial doses and research on plants along with revolutionary solutions to traditional issues.

**Fig. 1:**
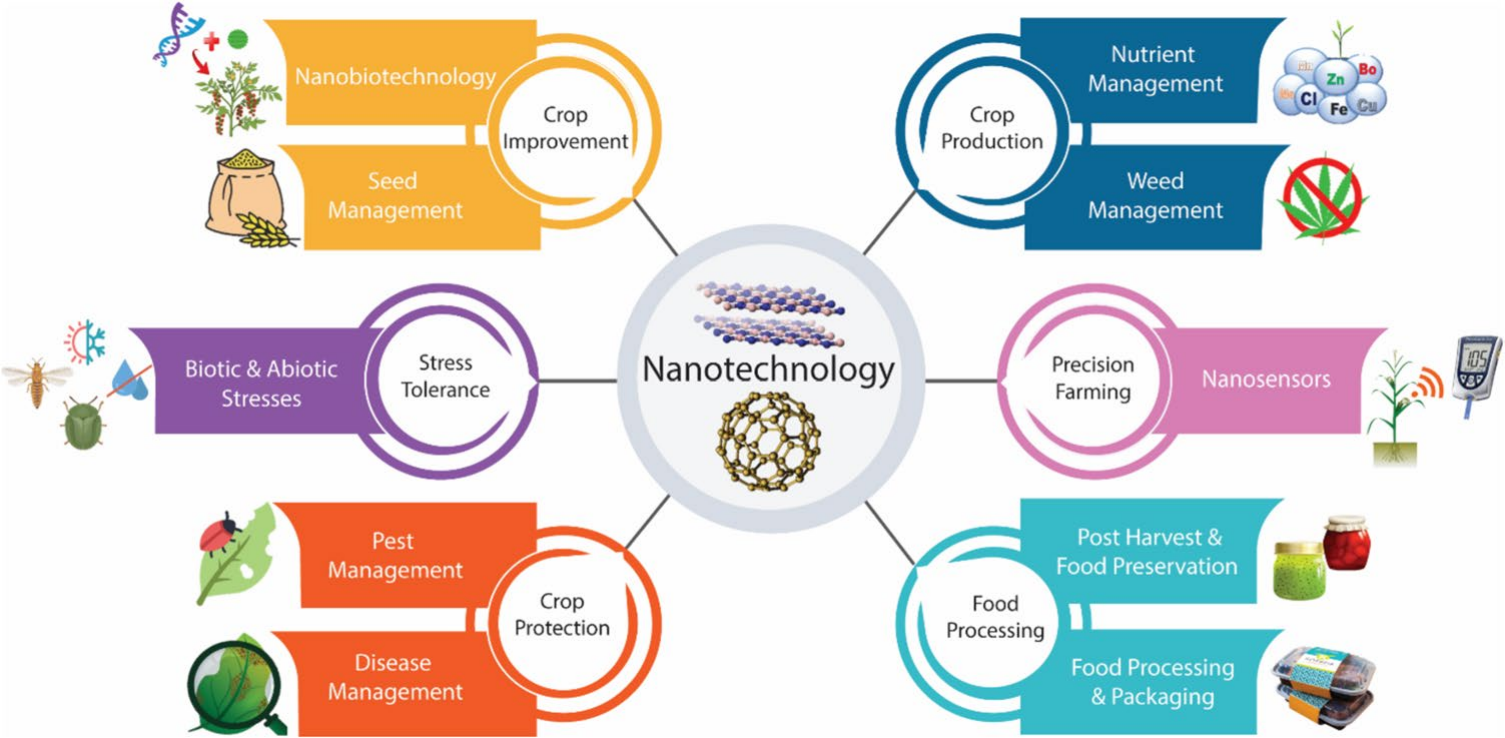
Potential applications of nanotechnology in agriculture. [Adapted from (Sharma 2023)].

In this context, the integration of nanotechnology with agriculture holds tremendous potential for addressing multiple facets of agriculture and food industry. Nanotechnology promises a paradigm shift by leveraging innovations such as enhanced disease diagnostics, improved plant nutrient uptake capacity, and other innovative domains. The emergence of nano-based crystals, currently under development, increases the efficacy of herbicides and insecticides even at substantially lower dosages. Moreover, the introduction of intelligent delivery systems and advanced sensors holds the potential to enhance agriculture’s resilience against viruses and diseases (Khan et al. 2019b; Gondal and Tayyiba 2022). Nanotechnology has the potential to contribute in various ways to improve agriculture (Fig. 1) (Sharma 2023).

Nanotechnology, with its ability to manipulate matter at nanoscale, high efficiency, robustness, and adaptable surface chemistry, can revolutionize various aspects of agriculture (Khan et al. 2019b; Lowry et al. 2019). Its transformative applications are illustrated by precision delivery systems for fertilizers and pesticides, nanomaterials for enhanced nutrient uptake by plants, and nanosensors for real-time monitoring (Hofmann et al. 2020). This precision-driven approach has the potential to not only optimize the utilization of resources but also minimize environmental impact, thus effectively addressing issues related to sustainability and ecological balance (War et al. 2020). Nanotechnology has revolutionized the era of precise farming by improving agricultural output, crop production, nutritional needs, and environmental protection (Malik et al. 2023).

**Fig. 2:**
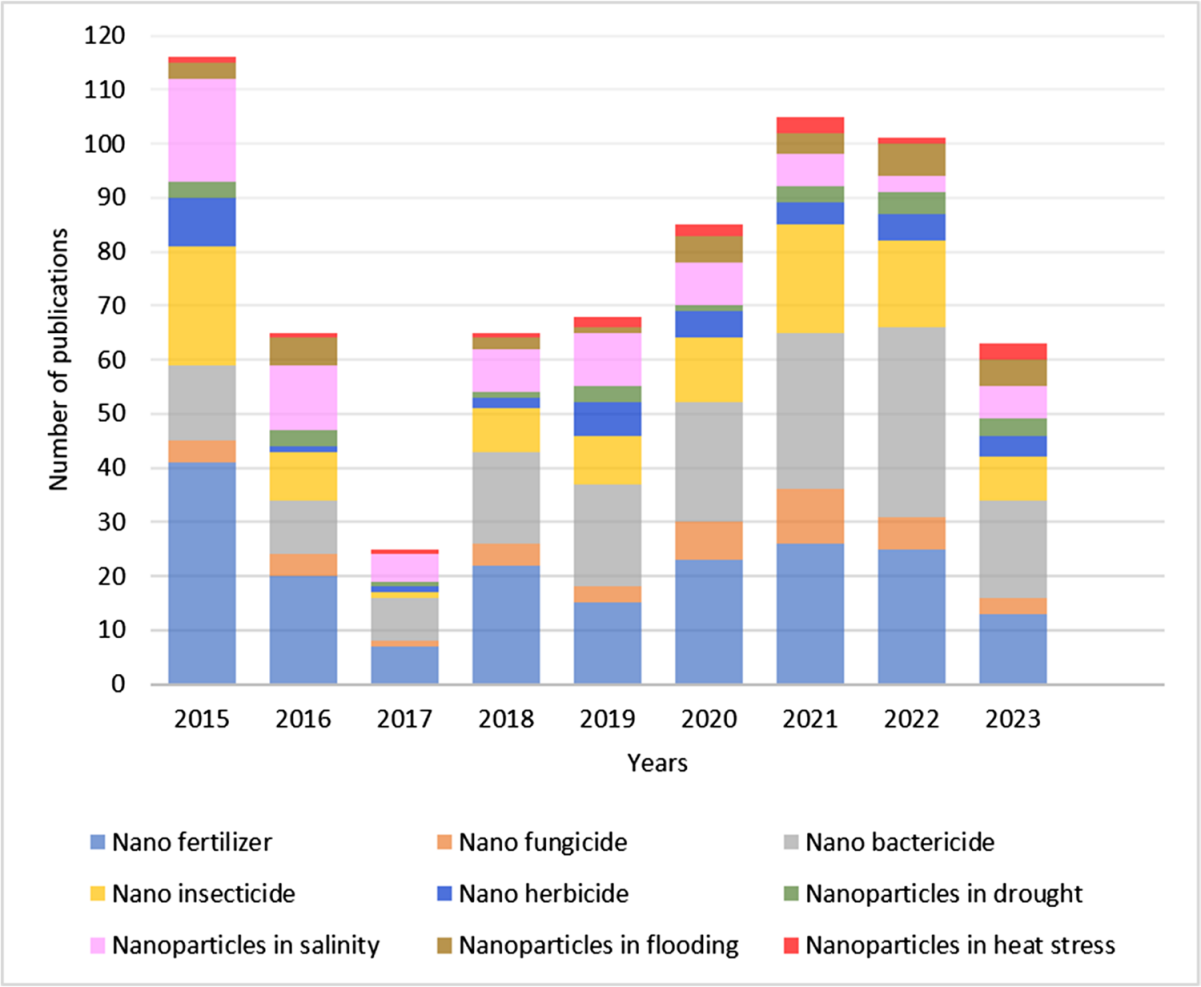
Articles published in PubMed regarding the application of nanotechnology in agriculture. The information was captured by applying the filters “Meta-Analysis, Research Supports” and the keywords mentioned above in legend with respective colors related to articles published since 2015

In the nanotechnology field, about 2356 companies from 60 different countries have developed 8918 products. Out of these, 226 products of agriculture sector were applied as fertilizer, plant breeding, crop husbandry, and plant breeding from 71 companies in 26 countries (StatNano 2022). The illustration of research progress in the application of nanoformulations for soil improvement, plant growth and development as well as to cope the biotic and abiotic stresses is depicted in Fig. 2.

Nano-based technologies encompass nano fertilizer, nano pesticides and nanosensors. These innovations have primarily found applications in agricultural practices and food quality enhancement (Ahmad et al. 2022). Chemical fertilizers have been considered an essential component for boosting crop yields since the green revolution, but their use has been linked with adverse environmental and ecological impacts. The leaching and gaseous emissions caused by the loss of nutrients from agricultural fields are major contributors to environmental pollution and climate change. However, nano fertilizers have emerged as a potential solution, helping to enhance the plant nutrients (Chandra et al. 2021). Application of nano zinc oxide (ZnO), iron oxide (FeO), and magnesium oxide (MgO) fertilizers in *Caesalpinia bonducella* enhanced the growth, chlorophyll contents, and nutrients by applying the dose of 100 ppm for Zn and 40 ppm for Fe and Mg (Khalid et al. 2022). Nano pesticides are useful in reducing environmental pollution, as well as controlled release and delivery of organic and inorganic ingredients to plants (Sharma et al. 2022b). Nanosensors have vast applications in numerous ways as diagnostic tools for pest identification, crop protection, soil condition detection, nutrient concentration, and water contents assessment (Thakur et al. 2022).

In addition to the alteration, identification, and detection process, nanosensors also have contributed to the precise farming by measuring seed viability and quantity of nutrients required for crops that may lead to maximizing yield with correct decisions (Mittal et al. 2022). The field of biotechnology has undergone a revolution owing to the introduction of tiny tools and gadgets with immense potential for a variety of uses. Nanotechnology has innovation in the biosensor field as nanoscale sensors provide remarkable sensitivity for the detection and quantification of pathogens, biomolecules, and environmental pollutants. Additionally, the development of quick and accurate DNA sequencing methods has been assisted by nanotechnology. Nanopore-based sequencing technology having the capability for single-molecule DNA analysis has enabled cost-effective high-throughput sequencing (Laszlo et al. 2014). The major limitation of Clustered Regularly Interspaced Short Palindromic Repeats (CRISPR) genetic engineering in plants includes the simultaneous insertion of CRISPR elements and donor template through homology-directed repair (HDR), cell wall that hinders the delivery of CRISPR reagents and time-consuming somatic transformation in plants that are not plausible for genetic engineering (Demirer et al. 2021).

Integration of nanotechnology with biotechnology has facilitated favourable miniature tools needed to modify plants for crop improvement compared to traditional tools and methods (Yan et al. 2022). Nanotechnologies have the potential to create safe and sustainable crops with improved stress tolerance, enhanced growth, nutritional quality, and yield (Zhao et al. 2022b). The NPs have greater efficiency, the ability to transfer various biomolecules, a positive correlation with the delivered genes, and suborganelle localization (Demirer et al. 2019b, c). Nanomaterials and devices have enabled characterization and precise manipulation of biological materials thereby revolutionizing the field of genetic engineering. The objective of this review is to explore the potential of nanotechnology and genomics in revolutionizing agriculture and promoting the sustainable development. Specifically, it aims to assess the impact of nanomaterials on crop growth, production, soil health improvement, coping with abiotic and biotic stresses, and nutrition. The innovations in genetic engineering for precise genome editing in plants and genomics with the integration of nanotechnology are also discussed.

## Role of nanotechnology in precision agriculture

Around the world, plant diseases lead to the annual loss of 20–40% of crops (Neme et al. 2021). Scientists have aimed to develop crop varieties with desirable traits in terms of both quality and quantity. Nanotechnology has offered innovative concepts in the field of pesticides, resulting in reduced environmental harm, longer shelf life, and improved water solubility (Chand Mali et al. 2020). Modern nanotechnology has demonstrated its capacity to alter the genetic makeup of crop plants, thereby aiding in the advancement of crop improvement over a substantial period (Squire et al. 2023). Throughout the course of agricultural revolution, natural and induced mutations have played a significant role in enhancing crop development (Al-Ebaidy et al. 2022; Shabani et al. 2022). The role of nanotechnology in minimizing postharvest losses and improving food processing is significant. Nanotechnology’s application to enhance shelf life, minimize spoilage, and improve food security is a critical trend that can address global food demands. The future of postharvest technologies is likely to focus on nano-enabled solutions for smarter packaging, real-time monitoring, and controlled release of preservatives (Neme et al. 2021).

Nanotechnology has introduced a new perspective to induced mutation research, replacing the utilization of conventional chemical agents, such as Ethyl methanesulfonate (EMS) and Methyl methanesulfonate (MMS), as well as physical mutagens like x-rays, gamma rays. The Nuclear Physics Laboratory (NPL) at Chiang Mai University in Thailand has harnessed nanotechnology to create a new white-grained rice variety derived from the traditional, purple-colored rice variety known as Khao Kam (Ganesh 2016). This entails the utilization of mutation breeding by Chiang Mai researchers. Their efforts are centered on determining the optimal route through a plant’s membrane and cell wall so that the nanoparticles (NPs) can penetrate the cell and induce a targeted alteration in the genetic makeup without interfering with other vital functions of cell wall and membrane (Pramanik et al. 2020). Since the inception of this technology, the United Nations, Food and Agriculture Organization (FAO) and International Atomic Energy Agency (IAEA) Programme in Vienna have made substantial contributions through mutation breeding and nuclear physics (Kharkwal 2023). The NPs play a role in plant improvement, as outlined in the comprehensive Table 1 (Gangwar et al. 2023). Conclusively, integration of nanotechnology into agriculture holds a solution for plant growth and development, and offers precise genetic modifications and sustainable crop enhancement. These efforts lead to improved agricultural practices and creation of novel crop varieties.

**Table 1:** Role of NPs in plant growth

NPs	Application for Plant Growth	References
Al_2_O_3_	Enhanced the root length of plants	(Farooqui et al. 2016)
Ag	Increased growth of the plant	(Sadak 2019)
Ag	Promoted growth and N fixation in chickpea (*Cicer arietinum*)	(Sambangi and Gopalakrishnan 2023)
Au	Increased the germination seed of *Arabidopsis thaliana*	(Kaushal 2018)
CaO	Significantly enhanced germination, seedling vigour, and root/shoot length in rice (*Oryza sativa*)	(Gopinath et al. 2023)
CeO	Enhanced fresh weight, dry weight and length of roots in cotton (*Gossypium hirsutum*)	(An et al. 2020)
CeO_2_	Induced oxidative stress in cucumber (*Cucumis sativus*) roots	(Xie et al. 2022)
CNTs	Improved germination and seedling biomass in mustard (*Brassica juncae*) and black gram (*Phaseolus mungo*)	(Ghodake et al. 2010)
CNTs	Increased vegetative growth by 1.5-fold and enhanced root length up to 202% at 20 µg/ml in *Brassica juncae*	(Mondal et al. 2011)
CNTs	Separated soil contaminants from water, boosted seedling growth, and extended root length	(Kaushal 2018)
CNTs	Improved germination index, germination percentage, number of leaves, and biomass in brinjal (*Solanum melongena*) and wheat (*Triticum aestivum*)	(Kothari Chhajer 2018)
Cu	Enhanced plant growth and yield	(Van Nguyen et al. 2022)
Fe	Improved the growth including leaf length, number of leaves, and shoots	(Arafaa et al. 2023)
FeO	Separate heavy metals from pollutants in soil	(Zhou et al. 2021)
GO	Germination of plants	(Sanzari et al. 2019)
SiO_2_	Elongated internodes by 27.4% and enhanced tillers by 66.7% in wheat (*Triticum aestivum*)	(Li et al. 2023)
ZnO	Enhanced phosphorous supplementation in cotton (*Gossypium hirsutum*) crop	(Venkatachalam et al. 2017)
ZnO	Synergic effect of PGPR improved plant growth and rescued from heat and drought stress in wheat (*Triticum aestivum*)	(Azmat et al. 2022)
ZnO rods	Increased growth of broccoli (*Brassica oleracea*) plant	(Kaushal 2018)
ZnO + Melatonin	Improved chlorophyll contents, Cd, Fe uptake, and growth in wheat (*Triticum aestivum*)	(Chen et al. 2023)

Aluminum oxide, Al_2_O_3_; Cadmium, Cd; Calcium oxide, CaO; Carbon nanotubes, CNTs; Ceric oxide, CeO_2_; Cerium oxide, CeO; Copper, Cu; Gold, Au; Graphene oxide, GO; Iron, Fe; Iron(II) oxide, FeO; Plant growth promoting rhizobacteria, PGPR; Silicon dioxide, SiO_2_ ; Silver, Ag; Zinc oxide, ZnO

### Improvement of soil health

One of the main sustainable development objectives of the century is to ensure nutrient security, food availability, and sustainable agriculture. Therefore, it is crucial to utilize the benefits of nanotechnology to accomplish the feat by increasing the availability of nutrients to plants and reducing their losses on agricultural soils (Elemike et al. 2019). Application of nanotechnology in soil health includes crop protection and production, focusing on nano biosensors, nano pesticides, nano fertilizers, as well as nano-enabled soil remediation techniques (Usman et al. 2020). The soil health improved by utilizing NPs is described in Table 2 (Gangwar et al. 2023). The NPs have the potential to enhance soil remediation, microbial activity, water absorption, nutrient uptake, and plant growth (Predoi et al. 2020). For example, carbon nanoparticles (CNPs) improved the nutrient use efficiency (NUE), uptake, soil fertility and crop growth in corn (Zhao et al. 2021). Another study reported the enhanced growth of *Trifolium repens* resulted in promoted phytoremediation in cadmium (Cd) polluted soil by the co-application of titanium dioxide (TiO_2_) NPs and plant growth promoting rhizobacteria (PGPR) (Zand et al. 2020a). Similarly, co-application of TiO_2_ and biochar to *Sorghum bicolor* positively affected the plant growth and development of antimony (Sb) contaminated soil (Zand et al. 2020b). The use of carbon nanomaterials improved seed water uptake and absorption from the cell, reaching the leaves and shoot after passing through the seed coat (Omar et al. 2019). Furthermore, SiO_2_ enhanced water absorption leading to improved seed germination, growth, and yield in potato (Ali et al. 2021a).

**Table 2:** Role of NPs in soil improvement

NPs	Application for Soil Fertility	References
Ag	Mobility of NPs in soil along with the clay particles	(Servin et al. 2015)
Ag	Negatively influences mutual interaction between fungi and plants and deteriorates arbuscular rhizospheric soil	(Cao et al. 2017)
Ag	Improvement in the microbial community composition	(Montes de Oca-Vásquez et al. 2020)
Ag	Soil fertility enhancement	(Ameen et al. 2021)
CeO_*2*_	Impacts organic matter in soil	(Majumdar et al. 2016)
CeO_*2*_	Soil nutrient retention	(Servin and White 2016)
CuO	Improvement in soil N fixation	(Guan et al. 2020)
FeO	Impacts soil properties	(Claudio et al. 2017)
FeO	Deterioration of chlorpyrifos	(Das et al. 2020)
MNPs	Affects soil properties	(Dimkpa and Bindraban 2018)
Se	Promotes poly-microbial biofilms ultimately leading to improved soil fertility	(Gudkov et al. 2020)
SiO	Seed germination improvement	(Fayiga and Saha 2017)
Si	Rhizospheric microbiome improvement	(Rajput et al. 2021a)
TiO_2_	Promotes RuBisCO activity and improves photosynthesis	(Servin et al. 2015)
ZnO	Cultivation of wheat (*Triticum aestivum*) in acidic and alkaline soils improves Zn content by 20-fold	(Servin et al. 2015)
ZnO	Improvement in soil properties such as distribution of moisture, evaporation, and water permeation	(Sheteiwy et al. 2021)
Zn + N	Increase in macronutrient (N, P, K) and micronutrient (Mn, Cu, Fe, Zn), availability as well as microbial population	(Sharma et al. 2023)
ZVI	Actively eliminate hexavalent Cr metal from pollutant land	(Su et al. 2016)

Ceric oxide, CeO_2_; Chlorpyrifos, CPS; Chromium, Cr; Copper, Cu; Copper(II) oxide, CuO; Iron, Fe; Iron(II) oxide, FeO; Manganese, Mn; Metal-based nanoparticles, MNPs; Nitrogen, N; Phosphorous, P; Potassium, K; Ribulose bisphosphate carboxylase/oxygenase, RuBisCO; Selenium, Se; Silicon, Si; Silicon monoxide, SiO; Silver, Ag; Titanium dioxide, TiO_2_ ; Zerovalent iron, ZVI; Zinc, Zn; Zinc oxide, ZnO

The NPs in soil health management have promising role in nutrient security and agricultural sustainability. Nano-enabled approaches showcase their potential to positively impact microbial activity, soil health, and nutrient availability thereby offering innovative solutions to mitigate environmental challenges.

### Nano fertilizer

Small molecules with a size range of 1–100 nm are called NPs and have physiochemical properties different from those of bulk materials (El-Saadony et al. 2020). Some nutrients are provided by nano fertilizer in a nano form, enhancing plant growth and productivity. Nano fertilizers give proper nutrients for promoting plant growth and soil applications, provide sustainable sources of plant nutrients, and high fertilization efficiency (El-Saadony et al. 2021).

One of the highly designed inputs that have been identified and shown to be reliable is nano fertilizers (Dutta and Bera 2021). Nano fertilizers are synthesized or modified versions of conventional fertilizers, bulk materials, or extracted from various vegetative or reproductive parts of the plants by various chemicals, physicals, mechanical, or biological methods empowered by nanotechnology. They are used to improve soil fertility, productivity, and the quality of agriculture yields. Notably, the bulk materials can be harnessed to synthesize NPs (Qureshi et al. 2018). The vast surface area of nano fertilizer coupled with particle sizes (1–100 nm) smaller than the pores in the plant’s leaves and roots allow for more penetration into the plant from point of application, leading to increased uptake of nutrients and improved efficiency in nutrient utilization (Singh 2017). Some nutrients have been provided by nano fertilizers at nanoscale, promoting plant growth and overall productivity (Gangwar et al. 2022; Singh et al. 2023c,). In addition to its nutrient delivery advantages, nano fertilizer offers many other benefits such as increasing crop yield, improving soil quality, reducing the use of chemical fertilizers and pesticides, increasing the water retention capacity of the soil, inhibiting weed growth, and enhancing biodegradability of organic waste (Yadav et al. 2023). Nanomaterial-coated fertilizer particles show higher surface tension than conventional fertilizer particles, resulting in more robust and efficient regulation of nutrient release (Bratovcic 2022).

**Fig. 3:**
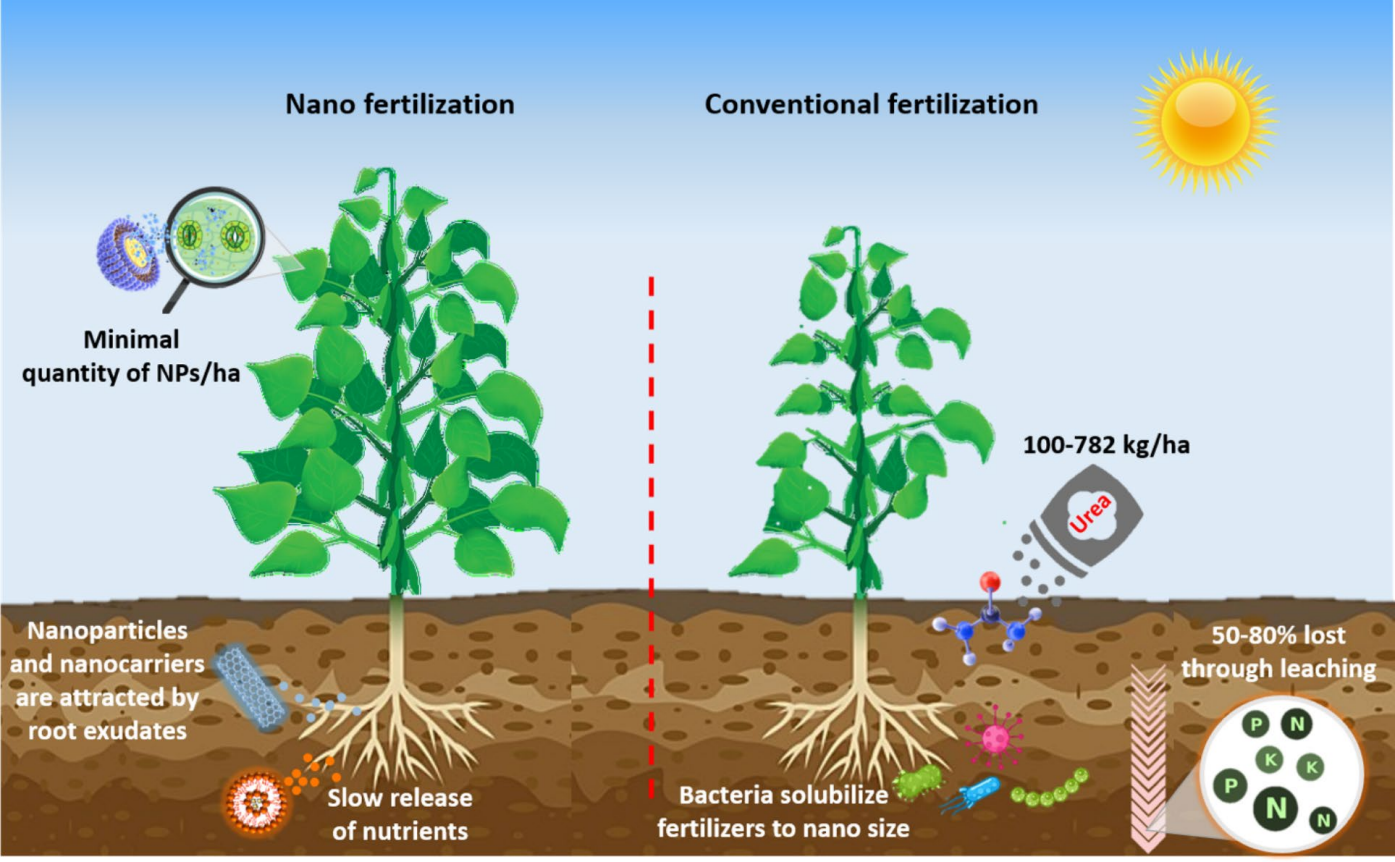
Effect of nano fertilizers and conventional fertilizers on plant and soil. [Modified from (Avila-Quezada et al. 2022)].

Nano fertilizer is divided into three distinct groups based on the nutrient requirements of plants macro nano fertilizer, micro nano fertilizer, and nanoparticulate fertilizers (Sahu et al. 2022). Macronutrient nanoformulations including N, phosphorus (P), potassium (K), calcium (Ca), and magnesium (Mg) are considered macro-nutrients and are coated on NPs to supply the exact amount of fertilizer to plants when needed (Basavegowda and Baek 2021; Khatri and Bhateria 2023). The efficiency and amount of macro nano fertilizer are reduced due to the high volume-to-surface ratio of NPs compared to conventional fertilizer. Many scientists have synthesized macro nano fertilizers and utilized them for healthy plant growth. In mungbean, MgONPs enhanced seed germination compared to conventional hydropriming (Anand et al. 2020). The N nano fertilizer reduced the nitrate leaching and improved sugar production in sugarcane (Alimohammadi et al. 2020). Nanoform of micronutrients that contain many of the minerals, such as magnesium (Mn), boron (B), silicon (Si), copper (Cu), Fe and Zn, but also include vitamins (C and B) improves the biomass and bioavailability of nutrients to plants (Khatri and Bhateria 2023). In squash, foliar application of manganese zinc ferrite (Mn_0.5_Zn_0.5_Fe_2_O_4_) enhanced the growth, biomass, number of rootlets, root and shoot length (Shebl et al. 2019). Application of CuNPs priming enhanced the response in maize under drought stress with the improvement in growth and yield (Van Nguyen et al. 2022). Similarly, chitosan-silicon NPs resulted in steady release and enhanced the growth and yield in addition to antioxidant defense enzyme mechanism in maize (Kumaraswamy et al. 2021). Moreover, ZnO nano fertilizer increased number of leaves, surface area, seed germination, root/shoot length, and seedling weight in broccoli (Awan et al. 2021). Nanoparticulate fertilizers, other than macro/macro nano ferttilizers, TiO_2_, SiO_2_, and CNTs also promote the germination and growth (Basavegowda and Baek 2021). The foliar application of fabricated TiO_2_ enhanced disease resistance and yield in *Capsicum annuum* (Prakashraj et al. 2021). Additionally, foliar application of SiO_2_, Se, ZnO and graphene nano fertilizers reduced the freezing injury in sugarcane (Elsheery et al. 2020b). The differential features of conventional and nano fertilizers are listed in Table 3; Fig. 3 [Modified from (Avila-Quezada et al. 2022)].

**Table 3:** The difference between nano fertilizers and conventional fertilizers

Properties	Nano fertilizers	Conventional fertilizers
Nutrient loss rate	Reduced fertilizer nutrient loss	More loss rate through leaching, run-off, and drifting
Controlled release	The release of pattern and release rate are controlled accurately	Nutrient release imparts soil toxicity
Solubility	High	Low
Bioavailability	High	Low
Mineral micronutrients dispersion	Enhanced dispersion of insoluble nutrients dispersion	Large-size particles lead to reduced solubility
Effective period of release	Effective and prolonged period	Utilized at the time and site of application; leftover is converted into an insoluble form
Nutrients uptake efficiency	Saves fertilizer via improved uptake ratio	Nutrient uptake efficiency is low due to the non-availability to roots
Soil adsorption and fixation	Reduced	High

Conventional fertilizers have low efficiency, for example, application of N fertilizer in soil results in a loss of up to 50–80% by the emission of nitrogen oxides and ammonia in gaseous form as well as leaching of nitrates (Yaseen et al. 2020). Additionally, the application of fertilizer decreased N by 40–70%, P by 80–90% and K by 50–90%, resulting in huge monetary losses (Dutta and Bera 2021). With the ability of greater efficiency, slow release of nutrients, providing crops with the exact amounts of nutrients, and reducing toxicity in soil, nano fertilizers can increase yield while maintaining environmental safety (Mejias et al. 2021). The application of nano fertilizer enhanced N utilization efficiency by up to 45% (Dutta and Bera 2021). Remarkably, improvement in the yield has been reported across various crops by utilizing the nano fertilizers (Table 4) (Iqbal 2019).

**Table 4:** Effect of nano fertilizers on the productivity of crops

Nano fertilizers	Crop Species	Increased Yield (%age)
Nano fertilizers + Urea	Rice	10.2
Nano fertilizers + Urea	Rice	8.5
Nano fertilizers + Urea	Wheat	6.5
Nano fertilizers + Urea	Wheat	7.3
Nano-encapsulated P	Maize	10.9
Nano-encapsulated P	Soybean	16.7
Nano-encapsulated P	Wheat	28.8
Nano-encapsulated P	Vegetables	12.0-19.7
Nano chitosan-NPK	Wheat	14.6
Nano chitosan	Tomato	20.0
Nano chitosan	Cucumber	9.3
Nano chitosan	Capsicum	11.5
Nano chitosan	Beetroot	8.4
Nano chitosan	Pea	20.0
Nano powder of cotton seed and ammonium	Cotton	16.0
Aqueous solution on non-Fe	Cereals	8.0–17.0
ZnONPs	Cucumber	6.3
ZnONPs	Peanut	4.8
ZnONPs	Cabbage	9.1
ZnONPs	Cauliflower	8.3
ZnONPs	Chickpea	14.9
Rare earth oxides NPs	Vegetables	7.0–45.0
AgNPs + Allicin	Cereals	4.0-8.5
FeONPs + CaCO_3_NPs + Peat	Cereals	14.8–23.1
S NPs + SiO_2_NPs + Synthetic fertilizer	Cereals	3.4–45.0

Calcium carbonate nanoparticles, CaCO_3_ NPs; Iron, Fe; Iron(II) oxide nanoparticles, FeONPs; Nitrogen, N; Phosphorous, P; Potassium, K; Silicon dioxide nanoparticles, SiO_2_ NPs; Silver nanoparticles, AgNPs; Sulfur nanoparticles, SNPs; Zinc oxide nanoparticles, ZnONPs

Revolution of agriculture with nano fertilizers offers controlled nutrient release, enhanced nutrient uptake, and improved solubility. Nano fertilizers dominate over conventional fertilizers by lowering toxicity in soil, reducing nutrient loss and availability of exact nutrient requirements thus highlighting their transformative impact on modern agriculture.

## Role of nanotechnology in conferring resilience to biotic and abiotic stress

Nanomaterials perform different mechanisms for the protection of plants but are challenged by several environmental stresses. Both biotic and abiotic stressors contribute to reduced product quality, plant growth, yields, and shelf life ultimately leading to economic losses. To counter these effects, the incorporation of beneficial insects, bacteria, and fungi onto seeds can enhance plant growth. Furthermore, the nano-formulations of insecticides, pesticides, bactericides, and fungicides, collectively known as agrochemicals, provide long-term plant protection effects which can reduce the use of chemicals on plants. These agrochemicals demonstrated low ecotoxicity and better yield and ecological balance (Gahukar and Das 2020). Another approach for protecting plants involves the introduction of NPs with insecticides, pesticides, bactericides, and fungicides, thus effectively protecting plants from various threats (Hazarika et al. 2022). Moreover, NPs can boost the secondary metabolism of plants, reinforcing their resilience against biotic and abiotic stresses (García-Ovando et al. 2022). However, the response of plants to these approaches relies on the factors such as the type of NP employed, plant species, and growth and development of the plants (Zulfiqar et al. 2019).

### Biotic stresses

Physical barriers, like stomatal pore closure and the waxy cuticle, are a plant’s primary line of defense against biotic stress and pathogens, but according to the type of pathogen including fungi, bacteria, and insects that may have mechanisms to pass these barriers (Silva et al. 2018). The PAMP-triggered immunity (PTI) is the first line response of plants to identify the pathogen including Ca and reactive oxygen bursts, transcriptional reprogramming, hormonal response, and mitogen-activated protein kinase (MAPK) signaling (Singh et al. 2022b). Various NPs have been developed that are beneficial for plant growth and development as well as mitigating the impact of biotic stresses (Table 5).

**Table 5:** Impact of various NPs in biotic stress

NPs	Target Crops	Biotic Stresses	Responses/Roles	References
Cu	Tomato (*Solanum lycopersicum*)	Bacterial	Developed tolerance against *Clavibacter michiganensis*	(Cumplido-Nájera et al. 2019)
MgO	Tomato (*Solanum lycopersicum*)	Bacterial	Induced systemic resistance against bacterial wilt	(Imada et al. 2016)
MgO + MnO_2_	Rice (*Oryza sativa*)	Bacterial	Application at primary growth stage promoted plant growth, improved photosynthetic activity, and reduced BLB expression	(Ogunyemi et al. 2023)
Ni + SiO_2_	Rice (*Oryza sativa*)	Bacterial	Suppressed the BLB disease	(Abdallah et al. 2023)
SiO_2_	Pea (*Pisum sativum*)	Bacterial	Inhibited 88.42% hatching and induced 43.79% mortality of *Meloidogyne incognita*	(Kashyap and Siddiqui 2021)
Ag	Tomato (*Solanum lycopersicum*)	Fungal	Increased total chlorophyll profile by 32.58% and fresh weight by 23.52% in tomato	(Kumari et al. 2017)
Ag	Onion (*Allium cepa*), Garlic (*Allium sativum*)	Fungal	Raised yield by 23.2% and 24.9% of onion and garlic, respectively	(Darwesh and Elshahawy 2021)
Chitosan	Rice (*Oryza sativa*)	Fungal	Suppressed rice blast fungus	(Manikandan and Sathiyabama 2016)
Chitosan + SA	Wheat (*Triticum aestivum*)	Fungal	Enhanced growth, activated transcription level, and protected from leaf rust	(Elsharkawy et al. 2022)
Cu	Tomato (*Solanum lycopersicum*)	Fungal	Enhanced the shoot biomass by 55.6% and suppressed the disease by 47.6%	(Cao et al. 2021)
Cu	Tomato (*Solanum lycopersicum*)	Fungal	Inhibited the Fusarium wilt by 66.5–68% and enhanced the chlorophyll content by 19.3–28.6%	(Lopez-Lima et al. 2021)
CuO	Tomato (*Solanum lycopersicum*)	Fungal	Chitosan-loaded CuONPs were effective against *Fusarium oxysporum* in reducing the disease	(Mosa and El-Abeid 2023)
Se + Ti	Tomato (*Solanum lycopersicum*)	Fungal	Reduced the severity by 20.4% and 41.5% by using Ti and Se, respectively	(Helmy et al. 2023)
Si	Oat (*Avena sativa*)	Fungal	Alleviated fungal stress and enhanced germination up to 80%, furthermore, increased relative water content, plumule, and radicle length	(Ahmad et al. 2023b)
SiO_2_	Watermelon (*Citrullus lanatus*)	Fungal	Reduced disease severity by 40%	(Buchman et al. 2019)
TiO_2_	Tomato (*Solanum lycopersicum*); Potato (*Solanum tuberosum*)	Fungal	Reduced mycelial growth by 90%	(El-Gazzar and Ismail 2020)
ZnO	Wheat (*Triticum aestivum*)	Fungal	Inactivated *Fusarium oxysporum* by 51.7%	(Zudyte and Luksiene 2021)
ZnO	Wheat (*Triticum aestivum*)	Fungal	Controlled leaf rust	(Badar et al. 2023)

Bacterial leaf blight, BLB; Copper, Cu; Copper(II) oxide nanoparticles, CuONPs; Magnesium oxide, MgO; Manganese dioxide, MnO_2_; Nickel, Ni; Salicylic acid, SA; Selenium, Se; Silicon dioxide, SiO_2_; Silver, Ag; Titanium, Ti; Titanium dioxide, TiO_2_; Zinc oxide, ZnO

#### Nano pesticides

Annually, almost four million tons of pesticides are deployed in crops leaving behind a potential threat to the environment and just a handful amount of 1–25% reach the target organisms (Zhang 2018). Moreover, ineffective pest and disease management is responsible for 20–40% of crop losses, ultimately reducing the crop economy by about US$220 billion annually (FAO 2019). The control of widespread pests in agricultural areas by conventional agriculture systems has induced increased pest resistance, disturbed soil biodiversity, and bioaccumulation of pesticides in the ecosystem. To address these environmental concerns, nanocarriers, known as smart delivery systems, can be utilized to achieve targeted delivery of the optimal active ingredients (Zhao et al. 2017a). In the pesticide sector, the main objective of nanotechnology is to lower the volume of pesticides to foster the stability of active ingredients, increase solubility, improve bioavailability, and enhance adhesion (Vasseghian et al. 2022). In 2018, the research on pest control effectiveness in the laboratory and field demonstrated that targeted nano pesticides are typically 24% more effective as compared to conventional counterparts (Kah et al. 2018). Therefore, one of the primary forces promoting sustainable agriculture is targeted nano pesticides. Consequently, the targeted nano pesticides are emerging as a catalyst for sustainable agriculture. The development of nano pesticides revolves around two methods: direct synthesis of nanosized pesticides or loading of active ingredients in nanocarriers. The loading procedure is carried out by different techniques such as absorption, encapsulation, and entrapment or attachment (Zhao et al. 2017a).

Multifunctional nanoplatforms have been developed as smart pesticide delivery systems including mesoporous silica, porous inorganic material, graphene oxide (GO), clay, and polymer (Wang et al. 2019c; Mukarram et al. 2021). Nano pesticides are reported to be widely utilized against various plant diseases for the targeted release of organic and inorganic components (Gahukar and Das 2020). The Cu and Ag nano pesticides have shown remarkable effectiveness in reducing pest activities (Athanassiou et al. 2018). Lambda-Cyhalothrin/Silver nano pesticide has shown 37% more insecticidal activity on cotton leafworm, *Spodoptera littoralis*, as compared to pesticide treatment alone (Ahmed et al. 2019). Additionally, diacyl hydrazine-based nanoformulation enhanced pest control activity by utilizing diet and topical incorporation methods (Pandey et al. 2020). Recently, chitosan-based rotenone NPs reported pesticidal activity against *Solenopsis invicta*, causing damage to vegetable crops (Zheng et al. 2022b).

To summarize this, innovative solutions are required to cope with the detrimental effects of traditional pesticides on environment. Targeted nano pesticides provide eco-friendly qualities and greater efficacy. In order to address pesticide related environmental damage, active ingredient delivery also offers efficient and productive agriculture future.

##### Nano fungicides

Fungal pathogens pose a significant threat to both food security and the agriculture sector due to conventional fungicidal resistance. Nanotechnology has the best solution to cope with this issue by developing antifungal drugs composed of NPs. Notably, AgNPs exhibited a higher antifungal activity biosynthesized by *Malva parviflora* extract (Al-Otibi et al. 2021). Additionally, AgNPs have demonstrated the antifungal activities against *Stromatinia cepivora*, a pathogen responsible for causing white-rot disease in onion and garlic. The AgNPs were found to showcase fungicidal effect within the concentration range of 40 and 200 mg/L affecting the sclerotia germination and mycelial development (Darwesh and Elshahawy 2021). Moreover, AgNPs exhibited antifungal activity using *Aspergillus niger* (Guilger-Casagrande et al. 2019) and *Trichoderma harzianum* (Al-Zubaidi et al. 2019). Similarly, AuNPs possess wound healing and antifungal effects (Korani et al. 2021). The SiO_2_NPs have also exhibited the potential to mitigate fungal disease severity in watermelon. For example, mesoporous silica and chitosan-coated mesoporous SiO_2_NPs enhanced the innate defense response by lowering disease severity by 40% and 27%, respectively (Buchman et al. 2019). Moreover, CuNPs provide essential nutrition to the plant, whereas the conventional fungicide copper hydroxide impedes proper plant growth. Additionally, CuNPs inhibited the fungal activity by 66.5–68.0%, improved the chlorophyll content by 19.3–28.6%, and served as plant growth promoters (Lopez-Lima et al. 2021). The application of CuNPs has proven effective in inhibiting the development of *Fusarium oxysporum* by 47.6% within tomato stems, demonstrating their potential for use in agricultural protection strategies (Cao et al. 2021) and reducing the disease incidences by 51.7% (Zudyte and Luksiene 2021).

The NPs offer the breakthroughs in improvement of plant health by providing effective antifungal activity, and fungicidal resistance as well as managing environmental impacts. More research is needed to explore the potential of NPs to advance these promising strategies.

##### Nano bactericides

In tomato studies, CuNPs have been reported to demonstrate antibacterial activity, inducing modifications in the concentration of phenols, glutathione, lycopene, and β-carotene (Cumplido-Nájera et al. 2019). Similarly, MgO induced antibacterial activity against the wilt disease in tomato (Imada et al. 2016). Bacterial inactivity was reported in pea by the application of SiO_2_NPs. This effect was further complemented by foliar spray that resulted in remarkable 10% increase in chlorophyll and 9.7% enhancement in carotenoid level (Kashyap and Siddiqui 2021). A recent study was conducted to check the effect of bacterial leaf blight (BLB) disease in rice plants. The Ni-SiO_2_NPs composite increased the plant growth and vitality as well as lowered the severity of BLB by enhancing the apoptosis of bacterial cells up to 99.61% (Abdallah et al. 2023). Moreover, the treatment of plants with MnO_2_ and MgONPs protected the plants from BLB, enhanced photosynthetic activity, and promoted the seedling growth of rice plants. The foliar spray of MnO_2_ and MgONPs resulted in diseased leaf areas by 21.86% and 15.04% as well as decreased bacterial number by 71.47% and 77.78%, respectively (Ogunyemi et al. 2023).

Deployment of NPs showcases the antibacterial activity, vitality, resistance to diseases, and positive effects of biochemical concentration ultimately leading to enhancement of crop production.

##### Nano insecticides

Insecticides are useful in mitigating various species of insects, moreover, these may have a negative influence by impacting non-targeted living species and risking environment (Alsafran et al. 2022). Additionally, it may result in bioaccumulation which is a greater threat to plants and animals (Zhang et al. 2023). Different NPs likewise CuO, ZnO, SiO_2_, and Ag have been implemented to resolve this problem and have insecticidal properties (Deka et al. 2021). By using NPs to deliver the active chemical to the appropriate target at the precise concentration and time, pesticide delivery systems (PDS) have been developed for maximum biological efficacy. This approach not only mitigates the drastic effects on non-targeted organisms but also achieves targeted control over specific insects. The NPs serve as carriers to efficiently deliver insecticide molecules. The SiO_2_ gel NPs have been reported to have both direct and indirect effects in repelling or killing insect pests, including *Chrysoperla carnea* and *Aphis craccivora* (Thabet et al. 2021). The AgNPs treatment resulted in the mortality of larvae in *Tinea pellionella* and *Tenebrio molitor*, demonstrating their promising insecticidal properties (Rankic et al. 2021).

Nano insecticides provide the promising alternative by preventing risks to non-target organisms because of enabled targeted pesticide delivery systems. Ongoing studies may help to optimize NPs by offering insecticidal qualities and lowering undesired consequences.

##### Nano herbicides

In agriculture, weeds are a major threat hindering crop productivity, demanding effective elimination strategies (Pirzadah et al. 2019). Unfortunately, frequent use of herbicides stimulated the development of herbicide-resistant weeds. Despite resistance management techniques, such as integrated weed management, frequent herbicide use can disrupt the ecosystem and render these techniques ineffective. A viable solution lies in encapsulation of herbicide in polymeric NPs, offering an alternative approach that can reduce herbicide consumption by improving solubility, efficiency, and productivity rate. This approach can ultimately contribute to environmental safety (Pudake et al. 2019). If a smart delivery system is bound with active compounds there will be less use and superior efficacy of herbicides. The fabrication of glyphosate herbicide by coating on diatomite/Fe_3_O_4_ nanocarriers resulted in 99% efficacy against weed (*Cynodon dactylon*) as compared to bare glyphosate with efficacy of 96% (Nasrollahzadeh et al. 2019; Xiang et al. 2017). The NPs coated on herbicide enter the root system and hinder glycolysis, leading to the starvation and death of the target weed (Yata et al. 2018).

Various NPs like nano-clay, chitson, lipids, and alginate can be employed as nano bioherbicides. These NPs ensure the controlled release of active compounds, thereby effectively regulating their distribution. This approach improves the utilization rate while minimizing the loss of active components (Singh et al. 2021). Chitosan NPs coated with metabolites extracted from *Fusarium oxysporum* showed notable anti-herbicidal activity against weeds (Namasivayam et al. 2015). The use of AgNPs and carboxymethyl cellulose (CMC) can influence the breakdown of herbicides (Kanwar et al. 2019). Nanoemulsion is another way to deliver the herbicides effectively that are evenly distributed on the leaf surface enhancing the permeability of active compounds in weeds (Zainuddin et al. 2019). Nanoencapsulation of herbicides such as astrazin, triazine, and ametryn has demonstrated an improvement in plant absorption capacity by up to 84% (Yadi et al. 2018). Nanocapsules containing mint essential oil have shown herbicidal activity for weeds while exerting a mild effect on non-targeted crops (Taban et al. 2020). The improved distribution and controlled release may revolutionize weed control with the help of nano herbicides utilizing different NPs.

#### Uptake and delivery of nano pesticides

**Fig. 4:**
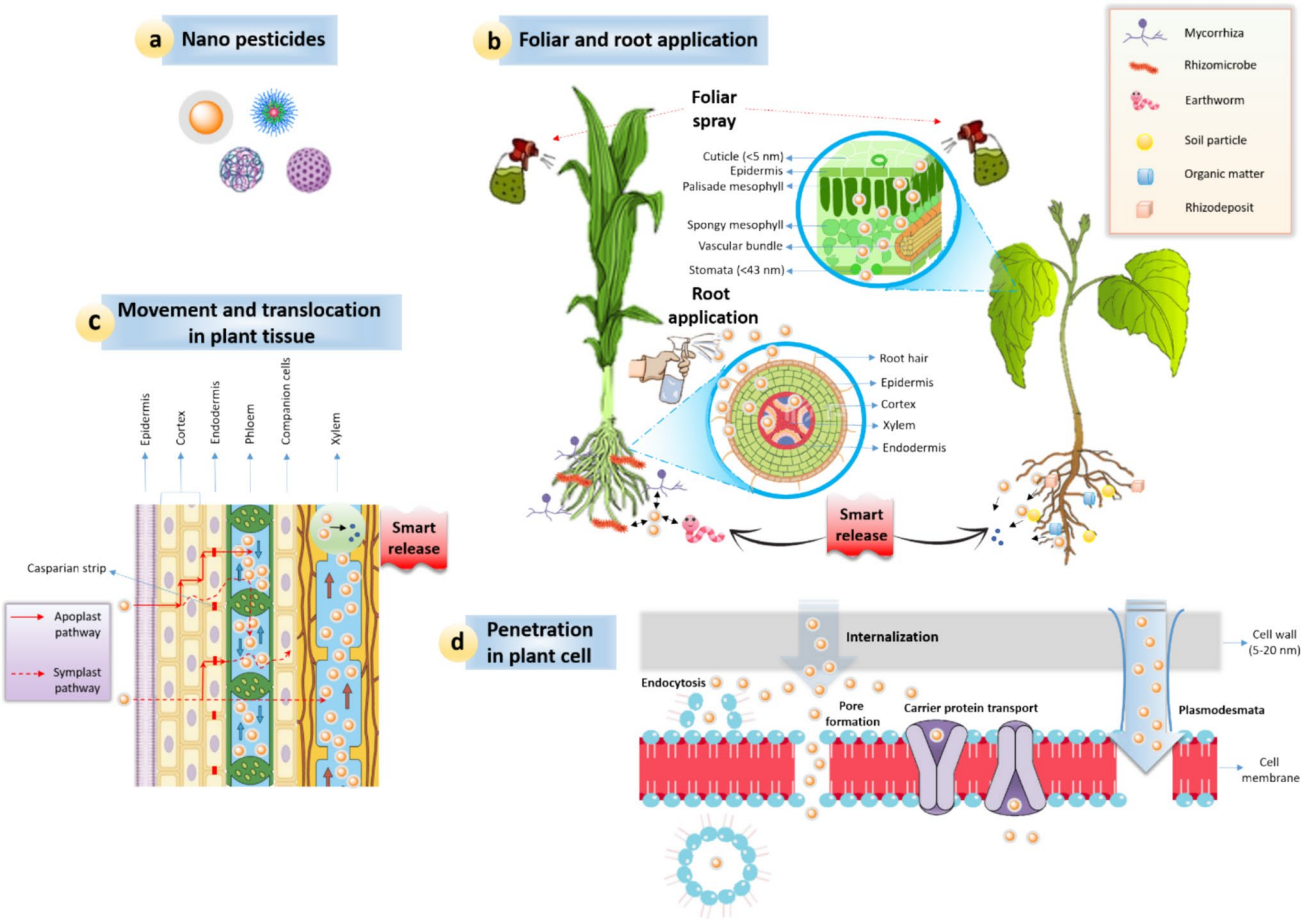
Schematic representation of plant-soil system showing nano pesticides’ uptake, translocation, and biotransformation as well as the influence on rhizosphere microbes. (**a**) Nano pesticides (type 2). (**b**) Foliar and soil application; opening in above-ground parts (stomata, cuticle, and shoot) provide the entry site for nano pesticides and transport them to lower portion via phloem. On contrary, the below-ground parts (root caps, lateral root junctions, epidermis, and cortex) facilitate the entry of nano pesticides from soil to plants and then translocate to upper parts through xylem. Rhizosphere microbiomes (fungi, bacteria, and archaea), rhizosphere invertebrates (worms, nematodes, and earthworms), symbiotic microbiota (rhizobia), rhizosphere deposits (plant root exudates), organic material and soil particles (sand, clay) have impact on uptake and translocation of nano pesticides. (**c**) Two important pathways, apoplastic and symplastic, are responsible to translocate the nano pesticides across various plant tissues. Smart release of active ingredients (AI) from responsive nanoscale delivery platform (RNDP) of nano pesticides improves adhesion and maintains long-term concentration against pests. (**d**) Internal pathways of piercing nano pesticides in plant cells involve endocytosis, pore formation, carrier protein and plasmodesmata. [Source: (Wang et al. 2022a)]

To improve the efficiency of nano pesticide utilization and minimize the losses, achieving strong adhesion and deposition of nano pesticides onto foliar surface is important (Yu et al. 2017). The NPs can enter in tissues of plants via shoot or root with the aid of foliar spray and root application, respectively (Ali et al. 2021c) (Fig. 4a). The NPs have more surface reactivity and large surface area and thus can deposit on epidermis of plant by hydrophobic affinity, adhesion and electrostatic adsorption (Achari and Kowshik 2018). For example, the tomato phloem and mesophyll tissues may quickly absorb 14–32 nm sized nano bactericides by depositing on cuticle and epidermis (Zhang et al. 2020b). In weeds, hydathodes permit the 256–345 nm sized nano herbicides to directly enter into mesophyll and vascular tissues (Bombo et al. 2019). Biological barriers allow smaller NPs to enter into cells due to the size limited structures (5–20 nm) (Fincheira et al. 2020). Moreover, large nano pesticides (80–200 nm) can still enter cells because of the interaction with cell wall including endocytosis, pore formation, carrier proteins, or cracks at lateral roots (Li et al. 2020) (Fig. 4b).

Generally, the interaction of plants and nano pesticides typically involves three steps (Su et al. 2019). Firstly, NPs are aggregated or absorbed on plant surfaces including leaves, stem, and root (Fig. 4b). Secondly, NPs traverse into cuticle and epidermis tissues from where they move towards vascular bundles through apoplasts (extracellular spaces including xylem vessels and cell wall) or symplasts (sieve tube, plasmodesmata) (Fig. 4c). Lastly, NPs are migrated to other parts of the plants via vascular tissues (xylem and phloem) (Fig. 4d). Similarly, same features are present in leaves (wax crystals, trichomes and cuticle) and roots (mesophyll, vascular bundle and epidermis containing stomata) but the differences in morphology features of leaves and roots may cause hurdle in the entry of NPs (Su et al. 2019). The successful transport and delivery of NPs depends on several factors including, type of plant tissue, NP shape, and application method (Schwab et al. 2016). Notably, phloem transport mechanism is involved in the delivery of NPs from leaf to root (Raliya et al. 2016).

The structure of leaves possesses higher hydrophobicity thus creating the hurdle for pesticides to attach to them thereby leading to waste of pesticides. The modifications in NPs can attach active groups and improve the pesticides’ capacity to adhere to leaves by adding or changing charges. The NPs have better adhesion to leaves and stems that cannot affect washing by rainwater and extend their contact period and utilization efficiency when compared to conventional pesticides (Yin et al. 2023). In cotton leaves, the activity period of Cry toxin was reduced by rainwater scouring. In contrast, when the Cry1Ac produced by *Bacillus thurigiensis* was loaded with magnesium hydroxide (Mg(OH)_2_), it enhanced the death rate of pests by 75% and increased the adhesion of toxin to leaves by 59% (Rao et al. 2018). Similarly, polydopamine-coated GO enhanced the oxaproxim retention on plant leaves (Tong et al. 2018). Moreover, concave Janus carriers enhanced the retention and deposition of pesticides to leaves (Zhao et al. 2019).

Recently, there has been a growing interest in development of abamectin-based NPs. One study investigated three different functional abamectin poly NPs to increase foliar retention in cucumber. The degree of adhesion was regulated by altering the functional groups attached to the NPs and was largely dependent on functional group (Yu et al. 2017). In another approach, chitosan based NPs for avermectin was utilized with poly-γ-glutamic acid (γ-PGA) adhesive group. This modification significantly enhances the adhesion of NPs on the foliage surface (Liang et al. 2018a). Additionally, multifunctional avermectin coated with glycine methyl nanocarrier improved the uptake and delivery of avermectin in rice plants (Wang et al. 2018). These nanocarriers also interact with amino acid transporters, leading to increased phloem loading and cellular uptake (Wu et al. 2021). The above-mentioned findings report that nonsystemic avermectin biopesticides improve the uptake and delivery in plants. Furthermore, AuNPs conjugated with D-glucose were actively transported and serve as an important ligand for uptake and translocation of nano pesticides in tobacco (Jia et al. 2017).

Future studies based on interaction between plants and nano pesticides should also examine residual behavior, dynamic digestion, and toxicity of NPs in addition to the migratory path mechanism. The interaction mechanism of nano pesticides with plants needs to be extensively studied to prevent the threat affecting the human and animal health which may be caused by long term accumulation in plants (Kannan et al. 2023; Ijaz et al. 2023). Additionally, nano pesticides may lead to negative effects in plants, for example, CuO nano pesticides induced membrane damage, nutrient uptake alteration, and disturbed antioxidant pathways in soybean and wheat as well as Ag nano pesticide application were more phytotoxic to tomato (Xu et al. 2023). Furthermore, to facilitate the development of environment friendly and effective nano pesticides, it is important to explore the changes in the physiological and morphological features of plants before and after NPs treatment. Such investigations would assist in the optimization of pesticide dosages, selection of suitable nanosized carriers, and identification of key factors determining the penetration process of NPs in plants.

### Abiotic stresses

Annually, abiotic stresses (climatic constraints) such as drought, heat salinity, waterlogging, and lack of nutrient deficiency, resulted in 51–80% crop losses (Li et al. 2021a). Such stresses induce pervasive challenges to cellular machinery affecting biochemical, molecular, and physiological changes in plants (Mushtaq et al. 2020). Abiotic stresses compromise the plant’s defense mechanism and make it more prone to biotic stresses. In response to these challenges, NPs hold potential to improve the plant’s secondary metabolism, offering a way to alleviate both biotic and abiotic stresses (García-Ovando et al. 2022). Various studies reported the role of NPs to cope with the abiotic stresses (Table 6).

**Table 6:** Impact of various NPs on plants under abiotic stress

NPs	Target Crops	Abiotic Stresses	Responses/Roles	References
ZnO	Sorghum (*Sorghum bicolor*)	Drought	Improved 94% Zn in grains, enhanced K acquisition by 16–30%, and mitigated drought stress	(Dimkpa et al. 2019)
ZnO	Wheat (*Triticum aestivum*)	Drought	Increased 18–29% Zn contents in seeds and improved plant growth	(Dimkpa et al. 2020b)
ZnO	Wheat (*Triticum aestivum*)	Drought	Enhanced drought stress tolerance	(Kausar et al. 2023)
ZnO	Wheat (*Triticum aestivum*)	Drought	Promoted wheat performance and enhanced Zn uptake by 28%	(Dimkpa et al. 2020a)
ZnO	Tomato (*Solanum lycopersicum*)	Drought	Higher activity of ascorbic acid antioxidants and free phenols, moreover, enhanced biomass, SOD, CAT and reduced H_2_O_2_	(El-Zohri et al. 2021)
ZnO	Cucumber (*Cucumis sativus*)	Drought	Improved soluble sugars, total amino acids, proline, glycine, betaine, and decreased the ROS	(Ghani et al. 2022)
ZnO	Maize (*Zea mays*)	Drought	Upregulated melatonin formation and increased antioxidant behavior	(Sun et al. 2020b)
ZnO	Maize (*Zea mays*)	Drought	The combined effect of ZnONPs and abscisic enhanced root length, chlorophyll contents, carotenoid, and relative water content, furthermore, reduced leaf damage index and stomatal opening	(Fatima et al. 2023)
ZnO + SiO_2_	Potato (*Solanum tuberosum*)	Drought	Enhanced quality, yield, and mitigated water stress	(Seleiman et al. 2023)
Fe, Co, Cu, and ZnO	Soybean (*Glycine max*)	Drought	Fe, Co, Cu, and ZnO upregulated the expression of drought sensitive genes by 2.16, 2.03, 1.73, and 1.40-fold, respectively	(Linh et al. 2020)
Ag	Wheat (*Triticum aestivum*)	Heat	Enhanced leaf area (18.3–33.8%), root length (22.2–26.1%), and shoot length (6.6–7.5%)	(Iqbal et al. 2019)
Se	Cucumber (*Cucumis sativus*)	Heat	Improved proline, CAT, and POD by 41%, 29%, and 48%, respectively	(Shalaby et al. 2021)
ZnO + TiO_2_	Wheat (*Triticum aestivum*)	Heat	Improved antioxidant effect, seedling performance, and combat heat stress by increasing the activity of SOD and CAT	(Thakur et al. 2021)
Ag	Pearl millet (*Pennisetum glaucum*)	Salinity	Improved the height growth and defense system	(Khan et al. 2020)
Ag	Roselle (*Hibiscus sabdariffa*)	Salinity	Activated the antioxidant enzymes and improved the flavonoid and anthocyanin contents by 69% and 77%, respectively	(Sadat-Hosseini et al. 2022)
C	Lettuce (*Lactuca sativa*)	Salinity	Enhanced the seed germination by up to 35%	(Baz et al. 2020)
Fe_2_O_3_	Peppermint (*Mentha piperita*)	Salinity	Stress resistor	(Askary et al. 2017)
K_2_SO_4_	Alfalfa (*Medicago sativa*)	Salinity	Enhanced plant biomass by 72%	(El-Sharkawy et al. 2017)
SiO_2_	Potato (*Solanum tuberosum*)	Salinity	Improved the plant growth at 50 mg/L	(Gowayed et al. 2017)
SiO_2_	Strawberry (*Fragaria* × *ananassa*)	Salinity	Improved cuticular transpiration and proline contents by 54% and 81%, respectively	(Avestan et al. 2019)
SiO_2_	Cotton (*Gossypium hirsutum*)	Salinity	Improved the leaf area, plant height, and biomass by 5.37%, 7.68%, and 43%, respectively	(Liang et al. 2023)
Si	Soybean (*Glycine max*)	Salinity	Alteration in antioxidant activity	(Farhangi-Abriz and Torabian 2018)
ZnO/Si	Mango (*Mangifera indica*)	Salinity	Improved resistance and production by 57%	(Elsheery et al. 2020a)
ZnO	Wheat (*Triticum aestivum*)	Salinity	Improved chlorophyll contents, boosted yeild and physical attributes	(Adil et al. 2022)
ZnO	Alfalfa (*Medicago sativa*)	Salinity	Enhanced salinity tolerance in addition to shoot and root growth by 29% and 28.4%, respectively	(Hassan et al. 2023)
ZnO	Maize (*Zea mays)*	Salinity	Mitigated salinity stress and significantly enhanced antioxidants, photosynthetic, and yield related traits	
ZnO	Rice (*Oryza sativa*)	Salinity	Enhanced root length, fresh and dry weight, enzymatic activity, and K^+^ content	(Singh et al. 2023a)
ZnO	Eggplant (*Solanum melongena*)	Salinity	The combination of ZnONPs and melatonin improved the total soluble proteins, total soluble sugars, photosynthetic pigments, and total free amino acids	(Anwar et al. 2023)
Al_2_O_3_	Soybean (*Glycine max*)	Flooding	Improved scavenging activity	(Mustafa and Komatsu 2016)
Si	Rice (*Oryza sativa*)	Heavy metal	Alleviated Cr toxicity	(Sharma et al. 2022a)
Si	Tomato (*Solanum lycopersicum*)	Heavy metal	Improved oxidative damage in shoot and root and significantly reduced its accumulation	(Yan et al. 2023)
ZnO	Wheat (*Triticum aestivum*)	Heavy metal	Enhanced dry weight and Zn content in plants while significant lowered the Cd accumulation	(Usman et al. 2023)

Aluminum oxide, Al_2_O_3_; Cadmium, Cd; Carbon, C; Catalase, CAT; Chromium, Cr; Cobalt, Co; Copper, Cu; Hydrogen peroxide, H_2_O_2_ ; Iron, Fe; Iron(III) oxide, Fe_2_O_3_; Peroxidase, POD; Potassium, K; Potassium ion, K+; Potassium sulfate, K_2_SO_4_ ; Reactive oxygen species, ROS; Selenium, Se; Silicon, Si; Silicon dioxide, SiO_2_; Silver, Ag; Superoxidedismutase, SOD; Titanium dioxide, TiO_2_; Zinc, Zn; Zinc oxide, ZnO

#### Effect of nanomaterial on heat stress

A study on heat stress suggested the association with yield reduction of 2.9%, 5.6%, 7.1%, and 10.6% for wheat, rice, maize, and soybean, respectively (Wang et al. 2020b). The SeNPs have demonstrated their potential in mitigating heat stress effects in various plants. For instance, in sorghum, the utilization of SeNPs was reported to enhance the growth, hydration, and chlorophyll content of tomato plants under heat stress (Djanaguiraman et al. 2018). Similarly, the foliar application of SeNPs application under high-temperature stress enhanced the proline level, relative water content, POD (peroxidase), and CAT (catalase) activities in cucumber plants (Shalaby et al. 2021). In case of wheat, AgNPs synthesized using the leaf extract of *Moringa oleifera* alleviated the heat stress by influencing the morphological growth of the plants (Iqbal et al. 2019). In mungbean, ZnONPs alleviated heat stress by improving physiological and biochemical attributes (Kareem et al. 2022). To sum up, the negative impacts of heat stress on crop productivity emphasize the dire need to figure out promising solutions for mitigation. The investigations mentioned above highlight the promising role of NPs in improving the adverse effects of heat stress on various crops, thus contributing to the development of strategies for enhancing crop resilience in the face of climate challenges.

#### Salinity stress and NPs

Increased population and reduced water resources force the farmers to use saline water for irrigating crop plants. Unfortunately, salinity stress negatively affects the biochemistry and physiology of plants, posing a greater threat to food security and crop production (Rossi et al. 2016). Epigenetic regulations are an area poised to revolutionize agriculture. Advancements in epigenetic mechanisms, particularly under plant stress, show promising potential in cereals, where external factors like drought and heat can now be better managed through these epigenetic changes. This could lead to stress-resilient crop varieties, significantly boosting productivity in cereals (Dinkar et al. 2024). In parallel, advances in nanotechnology are offering complementary solutions to environmental stresses, including salinity, drought, and nutrient deficiencies. Engineered NPs are being developed for use in agricultural soils and irrigation water, enhancing nutrient delivery and helping plants better withstand stress conditions.

In alfalfa, the physiological response was improved by the application of potassium sulfate (K_2_SO_4_) NPs due to the reduction of electrolyte leakage by 53%, enhanced proline content by 33%, a three-fold increase in catalase content, and 26% boost in antioxidant enzyme activity under salt stress (El-Sharkawy et al. 2017). The exogenous application of SiO_2_NPs at 50 mg/L mitigated the negative effects of the salinity in potato by increased activity of antioxidant enzymes (Gowayed et al. 2017). Seed germination and seedling growth were improved by the application of AgNPs in tomato plants (Almutairi 2016). Furthermore, salinity tolerance was reported in tomato plants (Alharby et al. 2017) and banana (Deepika et al. 2018) by the use of ZnONPs. In ajowan, the exogenous application of SA along with FeONPs adapted the plant to salinity stress by enhancing photosynthetic pigments, osmolytes, and antioxidant capacity (Abdoli et al. 2020). Application of ZnONPs mitigated the effects of salinity stress in wheat by increasing chlorophyll content (Adil et al. 2022). Recent studies reported that the application of ZnONPs improved physiological and biochemical indices in rice (Singh et al. 2022a) and sorghum (Rakgotho et al. 2022) and enhanced seed germination and enzymatic performance in maize (Alhammad et al. 2023; Ahmad et al. 2023a). Moreover, chitosan-MgONPs enhanced salinity tolerance in rice leaves (Song et al. 2023). The NPs coated with calcium phosphate (Ca_3_(PO_4_)) mitigated salinity effects, improved plant growth, activated oxidative stress indicators Malondialdehyde (MDA) and hydrogen peroxide (H_2_O_2_) and photosynthetic pigments in broad beans (Nasrallah et al. 2022). In tomato plants, SiNPs are responsible for alleviating salt stress and enhancing antioxidant activity (Alam et al. 2022). The CeONPs improved the fresh weight (41%), dry weight (38%), and seedling root length (56%) in cotton plants (An et al. 2020).

Conclusively, engineered NPs exhibit enhanced physiological responses and reduced biochemical changes due to salinity stress in a variety of plant species. With the goal to enhance plant resilience to salinity stress, nanotechnology can continue to expand NPs, utilization, investigate novel NPs and improve methods for delivery.

## Role of nanotechnology in environment sustainability and quality improvement

### Precision farming

Conventional farming practices excessively rely on agricultural inputs such as water, pesticides, machinery, and other inputs have resulted in severe environmental issues, including the release of greenhouse gases. The emergence of precision farming, also known as smart farming, attempts to address this issue by aiming to safeguard the environment and maximize the yield of crops (Klerkx et al. 2019). Precision farming can also help to reduce pollution, minimize expenditures and improve pesticide usage (Huang et al. 2018). Agriculture is being revolutionized worldwide by precision farming, which has many potential benefits for food safety, environmental protection, crop quality, sustainability, profitability, productivity, and rural economic development. Precision farming has grown to be an essential component to achieve these goals (Zhang et al. 2021). Due to the rapid increase in the cost of raw materials such as synthetic insecticides and fertilizers as a result of limited supply of natural gas and petroleum fuels, the importance of precision farming has even become more pronounced (Majumdar and Keller 2021). It serves as a promising alternative approach to enhance the yield and reduce the crop production costs (Prasad et al. 2017). However, introduction of precision farming may be challenging for some farmers. Not all will be able to deal with or may not accept the complicated processes such as computerized assessment of field conditions and subsequent data, which are crucial aspects of smart farming (Klerkx et al. 2019).

Precision farming combines sensors, computers, remote sensing devices, and global satellite positioning systems to collect real-time data as extensively as possible (Duhan et al. 2017). An excellent way to advance precision farming is through the use of nanosensors. In such scenario, nanosensors are used to quickly identify contaminations and pathogens that hinder crop growth and reduce crop production. To check the health status of plants, electronic devices incorporated nano based light emitting diodes are used to determine chlorophyll contents that significantly reduce pesticides and other agrochemicals (Acharya and Pal 2020). Several nano biosensors detected and measured viruses, bacteria, and pathogens for precision farming depending on the inhibition of enzymes or nanogenetics (Duhan et al. 2017). Nano biosensors have certain significant benefits over last generation biosensors (Antonacci et al. 2018). These provide the wise and sustainable utilization of chemicals (pesticides and fertilizer) and resources (water and land) with the real-time or continuous evaluation as well as traceability of factors responsible for production enhancement. Another benefit of nano biosensors is to work better in complex matrices like soil with accumulated chemicals and less homogenized soil that are responsible for background noise.

It is assumed that in near future precision farming will develop agricultural robots with nano biosensors which might serve as “decision support system” for farmers by robotically selective weeding (Acharya and Pal 2020). Nano biosensors are responsible for “sensing, monitoring and detection” of any biophysical or biochemical signal at a cellular or molecular level under stress conditions (Chand Mali et al. 2020). In order to truly recognize the potential advantages for environmental sustainability and global food security, this sector requires constant innovations and studies.

#### Quality enhancement/fortification

**Fig. 5:**
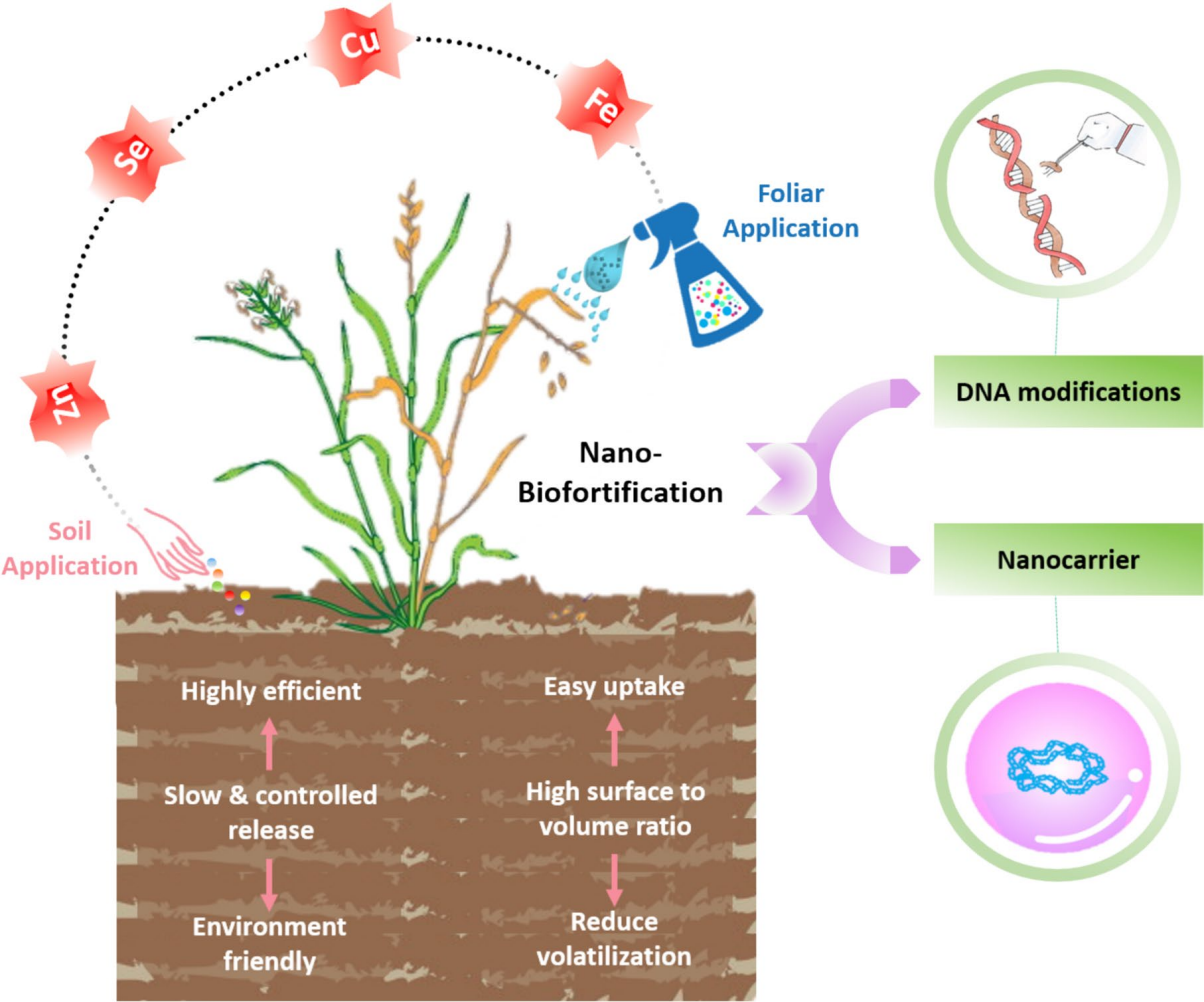
Biofortification of crops via foliar spray and soil application of Zn, Se, Cu, and FeNPs

Nanotechnology involves both the improvement of quality and quantity of crop plants in agriculture through biofortification (Xiong et al. 2017). Engineered NPs with desirable physical characteristics offer a viable solution for biofortification of food crops (Elemike et al. 2019). These NPs can boost growth, *in planta* micronutrient and crop output (root, shoot, and yield), through foliar spray or soil application (Ganesan 2015) (Fig. 5) [Source: (Kapoor et al. 2022)]. The NPs possess unique characteristics such as slow and controlled release mechanisms and high surface-to-volume ratios (Kabiri et al. 2017), which make them ideal candidates for addressing the micronutrient deficiencies in crops (Dimkpa and Bindraban 2018). Nano fertilizers supply nutrients more effectively than traditional agrochemicals, as they are required in small quantities. This precise delivery of NPs improves crop resilience, reduces nutrient losses through volatilization, boosts productivity, and even improves pesticide absorption in a sustainable manner. Innovations through newer technologies are driving value addition across the agri-food and nutrition sectors, offering potential breakthroughs in crop productivity, nutritional value, and sustainability. The development of value-added products utilizing integrated technologies will enhance the efficiency of agricultural production systems, ensuring a sustainable food supply chain (Kumar et al. 2018). Various NPs including Zn, Se, Cu, and Fe are most commonly used for quality improvement (Prasad et al. 2017). Detailed list of biofortified crops with micronutrient NPs are mentioned in Table 7.

**Table 7:** Role of various NPs to improve quality of crops

NPs	Target Crops	Application Methods	Enhanced Micronutrients	Other Positive Roles	References
Cu	Tomato (*Solanum lycopersicum*)	Foliar	Improved β-carotenoid, vitamin C, and Cu profile	Improved antioxidant response	(Hernández-Fuentes et al. 2017)
Cu	Sugarcane (*Saccharum officinarum*)	Soil	The Cu contents enhanced by 73%	Improved Cu, Mg, and Fe	(Tamez et al. 2019)
CuO	Wheat (*Triticum aestivum*)	Soil	Enhanced Cu contents by 18.84–30.45%	-	(Wang et al. 2019d)
CuO	Welsh onion (*Allium fistulosum*)	Soil	Enhanced Cu, Mg and Ca	Improved allicin profile in green onion by 6-187%	(Wang et al. 2020c)
CuO	Soybean (*Glycine max*)	Soil	Higher concentration of Cu by 1.8 times	Enhanced Cu concentration in seed, stem, leaf, and root	(Yusefi-Tanha et al. 2020)
Cu + Se	Tomato (*Solanum lycopersicum*)	Foliar	Enhanced vitamin C by 36%	Upgraded antioxidant response and yield	(Hernández-Hernández et al. 2019)
Fe(OH)₂	Wheat (*Triticum aestivum*)	Foliar	Increased Fe contents by 70–75%	-	(Zimbovskaya et al. 2020)
FeO	Wheat (*Triticum aestivum*)	Seed priming	Enhanced Fe contents by 26.8–45.7%	Improved root length and germination of seed	(Sundaria et al. 2019)
Fe_2_O_3_	Wheat (*Triticum aestivum*)	Soil	Enhanced Fe concentration	Enhanced biomass along with cysteine and tyrosine amino acids	(Wang et al. 2019d)
Fe_2_O_3_	Wheat (*Triticum aestivum*)	Hydroponics	Increased Fe uptake	Improved Chl a, Chl b, and carotenoids contents	(Al-Amri et al. 2020)
Fe_2_O_3_	Finger millet (*Eleusine coracana*)	Seed priming	Increased Fe profile up to 12.26%	-	(Chandra et al. 2021)
Fe_3_O_4_	Tobacco (*Nicotiana benthamiana*)	Foliar	Enhanced NPs uptake	Resistance against TMV and upregulated SA synthesis	(Cai et al. 2020)
Nano chelated Fe	Cumin (*Cuminum cyminum*)	Foliar	Increased Fe profile	Improved growth and yield of plant	(Sabet and Mortazaeinezhad 2018)
Se	Rice (*Oryza sativa*)	Foliar	Rice grains were enriched by Se up to 218.9–1096.6 µg/kg	Reduced Cd and Pb accumulation with increase in yield	(Wang et al. 2021)
Se	Rice (*Oryza sativa*)	Hydroponics	Improved Se profile by 6.7–20.4%	-	(Wang et al. 2020a)
Se	Cucumber (*Cucumis sativus*)	Foliar	Improved Se uptake	Improved plant growth and yield	(Shalaby et al. 2021)
Se	Coffee (*Coffea arabica*)	Foliar	Improved Se profile by 4.84–5.82 mg/kg	Enhanced the yield by 42%	(De Brito Mateus et al. 2021)
Se	Barley (*Hordeum vulgare*)	Seed priming	-	High germination at 5 mg/L	(Nagdalian et al. 2023)
ZnO	Wheat (*Triticum aestivum*)	Foliar	Enhanced Zn content in grain 27–35 mg/kg	Improved Zn profile in grains	(Zhang et al. 2017)
ZnO	Wheat (*Triticum aestivum*)	Foliar	Enhanced grain Zn profile	Improved seed germination and yield	(Doolette et al. 2020)
ZnO	Wheat (*Triticum aestivum*)	Foliar	Improved Zn concerntation by 30-fold in seed	Peroxidase and catalase activity was enhanced	(Sun et al. 2020a)
ZnO	Wheat (*Triticum aestivum*)	Foliar	Improved absorption of Zn	Protein and yield enhanced by 39.24%	(Sheoran et al. 2021)
ZnO complexed with chitosan	Wheat (*Triticum aestivum*)	Foliar	Enhanced Zn concentration upto 27–42%	Improved growth and yield of plant	(Deshpande et al. 2017)
ZnO	Wheat (*Triticum aestivum*)	Soil	Zn concentration increased in grains	Enhanced the yield by 56%	(Du et al. 2019)
ZnO	Wheat (*Triticum aestivum*)	Soil	Enhanced Zn concentration by 29%	Improved wheat performance	(Dimkpa et al. 2020b)
Urea coated ZnO	Wheat (*Triticum aestivum*)	Soil	Zn coated urea enhanced Zn uptake by 24% and uncoated by 8%	Coated and uncoated urea improved the yield by 51 and 39%, respectively	(Dimkpa et al. 2020a)
ZnO	Rice (*Oryza sativa*)	Foliar	Zn content enhanced by 37%	Yield improved by 42%	(Subbaiah et al. 2016)
ZnO	Rice (*Oryza sativa*)	Seed	Improved Zn profile	Enhanced antioxidant activity and total soluble sugar	(Sharma et al. 2021a)
ZnO	Rice (*Oryza sativa*)	Seed priming	Improved the total soluble protein by 0.37 mg/g	Enhanced the grain yield by 25.9%	(Adhikary et al. 2022)
ZnO	Rice (*Oryza sativa*)	Soil priming	Enhanced in Zn concentration by 13.5– 39.4%	Improved total biomass and NPK content 6.8–7.6%	(Yang et al. 2021a)
ZnO	Sorghum (*Sorghum bicolor*)	Soil	Improved Zn content by 94%	Increased N and P	(Dimkpa et al. 2019)
ZnO	Finger millet (*Eleusine coracana*)	Seed priming	Enhanced Zn concentration in grains by 13.96%	-	(Chandra et al. 2021)
ZnO	Mung bean (*Vigna radiata*)	Seed priming	Increased the Zn content by more than 4 600 µg/g	Positive effect on photosynthetic pigments and growth	(Sorahinobar et al. 2023)
ZnO	Potato (*Solanum tuberosum*) and Tomato (*Solanum lycopersicum*)	Foliar	Accumulated Zn in potato leaves and enhanced concentration in tomato leaves and fruit	-	(Singh et al. 2023b)

Cadmium, Cd; Calcium, Ca; Chlorophyll a, Chl a; Chlorophyll b, Chl b; Copper, Cu; Copper oxide, CuO; Selenium, Se; Iron, Fe; Iron hydroxide, Fe(OH)_2_; Iron(II) oxide, Fe_2_O_3_; Iron(III) oxide, Fe_2_O_3_ ; Lead, Pb; Magnesium, Mg; Nanoparticles, NPs; Nitrogen, N; Phosphorous, P; Potassium, K; Tobacco mosaic virus, TMV; Zinc oxide, ZnO

## Role of nanotechnology in genetic engineering

The NP-mediated delivery offers a favorable technique for plant genetic engineering, presenting a good alternative to conventional methods such as polyethylene glycol (PEG) treatment, Agrobacterium-mediated transgenic procedures, or gene gun bombardment (Wu and Li 2022). This innovative technique involves NPs to efficiently transfer genetic material into plant cells, streamlining the genetic modification process. Recent studies by Chandra et al. 2021 highlight how metal-based NPs, such as Zn and Fe, regulate the expression of key genes responsible for homeostasis in finger millet, a crucial cereal crop. The precise modulation of these homeostatic mechanisms opens doors for future genetic improvements in nutrient efficiency and stress resilience, enabling better yields under challenging environmental conditions. Compared to the traditional methods, NP-mediated delivery offers advantages, including enhanced efficiency, reduced complexity, and less disruption to plant tissues.

### Nanoparticles-mediated transformation efficiency

The cell wall in plants having a pore size limit of 5–20 nm serves as a major barrier to the transport of exogenous molecules (Zhang et al. 2019). Additional obstacles that must be overcome for the nuclear or plastid genomes to undergo genetic transformation include nuclear and organelle membranes in plant cells, which have significantly wider pore size limits of approximately 300–500 nm (Cunningham et al. 2018). A notable difficulty in genetic transformation involves the similarity in sizes of biolistic Au microparticles and micrometer-sized plant plastids, which renders it challenging to transfer DNA biolistically without destroying the organelle. The NP-medicated transformation presents a solution to these challenges, tackling issues such as low transformation efficiency and tissue damage to plants. This method can efficiently deliver the DNA across the cell wall, overcoming the barriers presented by plant structure (Lv et al. 2020).

Several reports indicated that biomolecule delivery via cell walls is possible without requiring any mechanical assistance like vertexing, ultrasound, electroporation, and biolistics (Table 8). Examples include: (1) Clay nanosheets delivered the RNA interference (RNAi) molecules to protect the tobacco plants against viruses (Mitter et al. 2017). (2) The magnetic iron oxide (Fe_3_O_4_) NP loaded into pollen allowed stable transformation to get diseased-free plants in cotton (Zhao et al. 2017b). (3) DNA nanostructures have been utilized for their capacity to carry short interfering RNAs (siRNAs) into the mature plant cells, leading to coordinated gene silencing in tobacco leaves (Zhang et al. 2019). (4) Several studies demonstrated the use of carbon nanotubes in model and non-model plants for the delivery of plasmid DNA (Demirer et al. 2019b, c; Kwak et al. 2019) and siRNA (Demirer et al. 2019a). Moreover, recent research reported the application of SiO_2_ in cannabis (Ahmed et al. 2021), ferroferric oxide (Fe_2_O_3_) in maize (Wang et al. 2022b), ferric chloride (FeCl_3_) in okra (Farooq et al. 2022) for the delivery of plasmid DNA and GO in tobacco protoplast (Li et al. 2022) to deliver siRNA. Thus, these NPs combined with new technologies provide promising solutions to advance plant biotechnology by getting transgenic plants that are stably efficient and transformed.

**Table 8:** The NP-mediated transformation in different plant species

NP Types	Targeted Tissues	Plant Species	Delivery Type	StableTrans-formation /Transient Expression	Transformation Frequency	Purpose	References
SWCNTs	Protoplast	Tobacco (*Nicotiana tabacum*)	Plasmid DNA	Stable transformation	8%	-	(Burlaka et al. 2015)
MWCNTs					3%	-	
Clay nanosheets	Leaves	Tobacco (*Nicotiana tabacum*)	dsDNA	Stable transformation	-	Protection against viruses	(Mitter et al. 2017)
Fe_3_O_4_	Pollen	Cotton (*Gossypium hirsutum*)	Plasmid DNA	Stable transformation	Approx. 1%	-	(Zhao et al. 2017b)
FeCl_3_	Embryo	Okra (*Abelmoschus esculentus*)	Plasmid DNA	Stable transformation	Approx. 1%	-	(Farooq et al. 2022)
Chitosan-complexed SWCNTs	Chloroplast	Arugula (*Eruca sativa*), Watercress (*Nasturtium officinale*), Tobacco (*Nicotiana tabacum*), and Spinach (*Spinacia oleracea*)	Plasmid DNA	Transient expression	Approx. 88%	-	(Kwak et al. 2019)
SWCNTs	Leaves	Tobacco (*Nicotiana tabacum*)	siRNA	Transient expression	95%	-	(Demirer et al. 2019a)
CNTs	Leaves	Tobacco (*Nicotiana benthamiana*), Arugula (*Eruca sativa*), Wheat (*Triticum aestivum*), and Cotton (*Gossypium hirsutum*)	Plasmid DNA	Transient expression	85%	High protein expression levels	(Demirer et al. 2019b)
CNTs	Leaves, Protoplast	Tobacco (*Nicotiana benthamiana*), Wheat (*Triticum aestivum*), Cotton (*Gossypium hirsutum*), and Arugula (*Eruca sativa*)	Plasmid DNA	Transient expression	85%	Strong protein expression	(Demirer et al. 2019c)
DNA nanostructures	Leaves	Tobacco (*Nicotiana benthamiana*)	siRNA	Transient expression	80%	Gene silencing	(Zhang et al. 2019)
SWCNTs	Roots	Tobacco (*Nicotiana tabacum*)	Plasmid DNA	Transient expression	-	-	(Golestanipour et al. 2018)
Au@SiO_2_	Leaves	Marijuana (*Cannabis sativa*)	Plasmid DNA	Transient expression	-	-	(Ahmed et al. 2021)
GO	Protoplasts	Tobacco (*Nicotiana tabacum*)	siRNA	Transient expression	97.2%	Gene silencing	(Li et al. 2022)
Fe_3_O_4_	Pollen	Maize (*Zea mays*)	DNA	Transient expression	29–74%	-	(Wang et al. 2022b)

Carbon nanotubes, CNTs; Ferric chloride, FeCl_3_; Graphene oxide, GO; Iron (II, III) oxide, Fe_3_O_4_; Multi walled carbon nanotubes, MWCNTs; Silicon dioxide-coated gold, Au@SiO_2_; Single walled carbon nanotubes, SWCNTs

### Nano-enabled precise genome editing

**Fig. 6:**
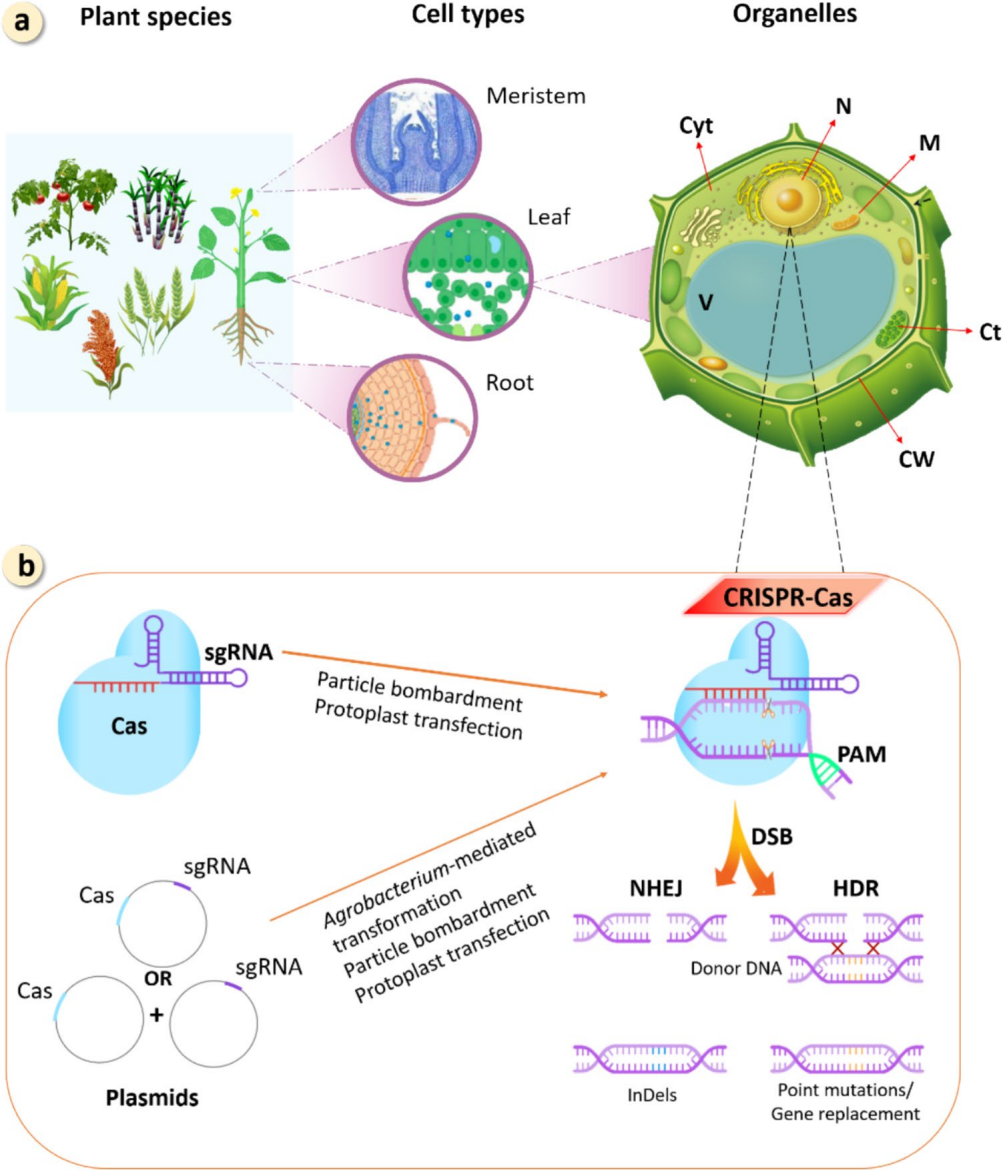
Delivery of CRISPR-Cas in cells and organelles of different plant species. (**a**) Target of CRISPR-Cas elements including cell types (meristem, leaf, and root) and organelles (nucleus). (**b**) Genome editing is carried out by delivering the CRISPR-Cas system into cells. The sgRNA guides the Cas protein to create double-stranded break (DSB). Two DNA repairing mechanisms are triggered; Homology directed repair (HDR) and Non-homologous end joining (NHEJ). Chloroplast, Ct; Cytoplasm, Cyt; Cell wall, CW; Mitochondrion, M; Nucleus, N; Vacuole, V; Single-guide RNA, sgRNA; Genomic RNA, gRNA; Protospacer adjacent motif, PAM. [Source: (Demirer et al. 2021)]

The emergence of precise and efficient plant genome editing technologies has created new opportunities for crop development and establishment of sustainable farming systems (Li et al. 2021a, 2024; Rahman et al. 2022). One of the most prominent technologies, CRISPR/Cas (CRISPR-associated protein), has transformed genome editing in crops, enabling precise genome modification of crops that lead to the development of stress tolerant and high yielding crop varieties (Sharma and Lew 2022). The CRISPR/Cas9 system coupled with nanotechnology has been widely used in livestock, and food industry and demonstrated widespread acceptability (Islam et al. 2020). The NPs are species-specific (Fig. 6), adding a layer of precision to genome editing approach. However, to fully explore the possibilities of CRISPR-mediated genome editing systems in plant biology, further advancements are needed in nanotechnologically specialized tools. Nanomaterials allow the CRISPR/Cas genome modifications to be organelle specific and have been applied for the identification of infectious disorders and the reduction of infection rates (Zafar et al. 2020a, b).

Nanotechnology coupled with CRISPR/Cas9 targeted gene editing technique could help us overcome many obstacles in agriculture industry. Nanotechnology assisted-CRISPR/Cas9 system may regulate gene expression for a specific characteristic based on gene function, resulting in development of resilient crops (Demirer et al. 2021). Nanomaterial-assisted CRISPR/Cas genome modifications have not yet been reported in plants because of the high delivery efficiencies and the distinct physicochemical properties of CRISPR reagents, despite the success of delivering genes and proteins into different cells of the plant by utilizing NPs for transgenics (Demirer et al. 2020; Yan et al. 2022).

A recent study by Demirer et al. (2021) has discussed in detail how nanotechnology is advancing CRISPR-Cas in plant genetic editing. The use of nanomaterials for plant genome editing is still challenging with numerous unresolved problems. Firstly, the amount and size of DNA and protein efficiently delivered to plants and surface chemistry of NPs compatible with plant tissues. Secondly, although the efficient delivery of some nanomaterials into plant chloroplasts, it is still unknown whether these nanomaterials can carry CRISPR elements to modify the genome of plants by using mitochondria and chloroplasts. Thirdly, the compatibility of nanomaterials with regeneration and tissue culture still requires more studies when genetic transformation is not applicable. Lastly, is there any change in plant regulation modified via nanomaterials and traditionally modified ones?

Keeping in view these issues, the adoption of this technology requires comprehensive understanding of delivery mechanisms, nanomaterial-plant interaction, developmental progress of NP chemistries, and health and environmental hazards.

The major contribution of nanotechnology in genetic engineering is the targeted delivery in cells and tissues of plant species. Mesoporous SiO_2_NPs with genetic material have been delivered through the cell wall into the cytoplasm of plant protoplasts and seedlings using the biolistic method (Torney et al. 2007). Certain NPs’ unique or engineered fluorescent features enable imaging of cargo delivery and release processes *in planta*, in addition to precise delivery, controlled release of cargo and protection of cargo from breakdown (Hu et al. 2020). DNA nanostructures triggered gene silencing in mature plants (Zhang et al. 2019) and delivery of clay nanosheets enhanced the resistance against pepper mild mottle virus (PMMoV) and cucumber mosaic virus (CMV) (Mitter et al. 2017). Moreover, clay nanosheets prevented the degradation of artificial micro-RNA (amiRNA) that targets tomato yellow leaf curl virus (TYLCV) by silencing the targets produced by virus and increasing the level of H_2_O_2_ (Liu et al. 2020b). Additionally, plasmids can be induced by NPs to penetrate plant tissues and enhance gene expression (Demirer et al. 2019a; Kwak et al. 2019). The genetic modification in cotton plants was conducted via magnetic NPs as carriers in the pollens (Zhao et al. 2017b). The goal of future studies must be directed to explore real-time imagining technologies, refinement of NPs for the improvement of delivery efficiency, and broaden the adaptability and genome editing in diverse plant species.

## Role of nanotechnology in genomics

### NanoPCR

The traditional PCR (Polymerase Chain Reaction) is associated with certain drawbacks including difficulty in amplifying GC-rich regions in genomic DNA and non-specific product amplification. To resolve this problem, one of the modified approaches termed as NPs-assisted PCR (nanoPCR) has undergone substantial research since 2004 (Tabatabaei et al. 2021). A variety of nanomaterials has been employed to improve the efficiency of nanoPCR such as CNPs, nanoalloys, quantum dots, liposomes, and metal NPs (Gao et al. 2019).

Integrating NPs into PCR experiments often enhanced DNA replication fidelity, improved PCR yield, boosted template DNA’s ability to bind to DNA polymerase and primers, as well as processed the thermal cycling mechanism. In contrast to inorganic NPs, which are usually added to PCR process as enhancers, liposome-based PCR is a unique kind of nanoPCR serving as hardware for the PCR assay in a nucleus or cell. Future directions must focus on tackling challenges involved in genomic DNA amplification by expanding and optimizing the range of NPs to improve PCR efficiency.

### ELISA

An enzyme-based immunoassay, the traditional enzyme-linked immunosorbent assay (ELISA), recognizes and enhances affinity interactions among antigens and antibodies with the help of enzymes by converting chromogenic substrates (Tabatabaei et al. 2021).

To overcome the drawbacks of traditional ELISA, NPs-based immunoassays have drawn a lot of interest because of their longevity, sensitivity, and cost-effectiveness (Singh et al. 2019). For example, by enhancing the signal strength and adding more binding sites for detecting antibodies, nanomaterials increased the detection ability of traditional ELISA assays (Billingsley et al. 2017). The quantification of immunoglobulin G enhanced specificity, sensitivity, and stability by employing Hemin-MnO_2_ nanocomposite as an enzymatic label in place of horse radish peroxidase (HRP) enzyme (Guo et al. 2017). Magnetic NPs modified ELISA (MELISA) assay showed significant signal amplification, source for magnetic enrichment, and improved the detection capability (Wu et al. 2017). Further research and integration of NPs in immunoassay technology is required to boost stability, specificity, and sensitivity. Future directions must be with the aim to wider and improved performance in diagnostic and healthcare.

### Nano biosensors

Conventional agricultural detection methods are typically expensive and labor-intensive approaches that require several sample preparation steps before detection, as well as complex instruments and trained workers that are not readily available, particularly for people living in remote regions (Mufamadi and Sekhejane 2017). In the agri-food industries, nano biosensors provide a cost-effective detection instrument with better sensitivity. The efficiency of sensors is greatly increased by adding NPs and nanostructures on the basis of portability, selectivity, multiplex detection capabilities, and sensitivity (Sharma et al. 2021b; Kumar et al. 2021). In biosensing research, a number of NPs have now garnered significant attention. Multiple analytes can be detected using biological probes combined with various NPs, including metallic, magnetic, carbon nanotubes, GO, and quantum dots (Thakur et al. 2022).

Nano biosensors enable quick screening of contaminants in water and soil at nanomolar to picomolar levels. Additionally, these are particularly useful for monitoring sustainable farming techniques for excessive fertilizer application and in the areas of food safety (Verma and Rani 2021). The low limit detection and the sensing ability have earned them significant recognition in agriculture farming (Sharma et al. 2021b; Mathivanan 2021). Due to their remarkable chemical, optical, and electrical properties, as well as excellent biological compatibility and low toxicity, ZnO and CNPs are multipurpose nanomaterials for the agricultural industry (Shojaei et al. 2019). The Se, platinum (Pt) and Ag are widely used metal NPs due to surface electric effect, catalytic and optical properties (Lau et al. 2017; Ahmed et al. 2020; Yu et al. 2021). Graphene-based biosensors are potentially useful due to their distinguishing features including oxygenated functional groups and high biocompatibility that help to create metal composites (Verma et al. 2022). Detection of pathogens, plant diseases, heavy metals, pesticides, phytohormones, and soil quality are the most crucial functions of nano biosensors (Ali et al. 2021b).

Plant pathogens are the main cause of declining crop yield, which could lead to a food shortage for humans as well as livestock. Globally, plant pathogens are thought to be responsible for 16% of crop productivity losses (Ficke et al. 2018). Scientists have devised various biosensing strategies including voltametric sensing, aptamer sensing, electrochemical sensing, photoelectrochemical sensing, immune sensing, etc. for pathogen and disease detection, physiological stress detection, toxic chemical ddetection, and heavy metal detection (Table 9). Some other electrochemical biosensors were also present that can detect the phytohormones under different stress conditions (Wang et al. 2020b; Cao et al. 2020; He et al. 2020).

**Table 9:** Nano biosensors utilized in agriculture

Type of Nano biosensor	NPs	Detection Limit	Specific Target	References
Pathogen and Disease Detection				
Chemiresistive	SWCNTs	5 nM	*Citrus Huanglongbing* (HLB)	(Tran et al. 2020)
	rGO and AuNPs	> 97%	*Phytophthora infestans* infection/tomato	(Li et al. 2021b)
Colorimetric	AuNPs	10^2^ CFU/mL	*Xanthomonas campestris*/brassica	(Peng and Chen 2019)
Optical	AuNPs	~ 100 cells	*Xanthomonas campestris*	(Peng and Chen 2019)
	AuNPs	0.4 ppm	*Phytophthora infestans*/tomato	(Li et al. 2019)
	Cysteine functionalized AuNRs/AuNPs	~ 0.4 ppm	*Phytophthora infestans*	(Li et al. 2019)
Lateral flow assay	CNPs	104 CFU/ml	*Xanthomonas arboricola* pv. *pruni*	(Lopez-Soriano et al. 2017)
	AuNPs	0.1 pg/µL	*Phytophthora infestans*	(Zhan et al. 2018)
	AuNPs	0.01 pg	*Alternaria panax* Whetz	(Wei et al. 2018)
	AuNPs	0.2 ng/mL	Potato leafroll virus (PLRV)	(Panferov et al. 2018)
Electrochemical	AuNPs	214 pmol/L	Plant pathogen DNA detection	(Lau et al. 2017)
	AuNPs	0.01 nM	*Xanthomonas axonopodis*/citrus	(Haji-Hashemi et al. 2018)
	AuNPs	0.3 fg/mL	CTV/sweet orange trees	(Freitas et al. 2019)
	AuNPs	337 CFU/mL	*Pseudomonas syringae* pv. *lachrymans*	(Cebula et al. 2019)
	AuNPs	100 nM	CTV	(Khater et al. 2019a)
	AuNPs	1 pg/µL	CTV	(Khater et al. 2019b)
	AuNPs	100 CFU/mL	*Escherichia co*li detection	(You et al. 2020)
	AuNPs	0.01 µM	Organophosphorus pesticide methyl parathion detection	(Zhao et al. 2020)
	GO	5.7 ng/mL	GBNV	(Chaudhary et al. 2021)
	Graphene	1.6 µM	Glyphosate, 2,4-dichloro phenoxy acetic acid and dicamba	(Kucherenko et al. 2021)
	Graphene	10 fmol/L	False smut result of *Ustilaginoidea virens*	(Rana et al. 2021)
	Graphene	Detection limits (CLO, 823 nM; IMD, 384 nM; TMX, 338 nM; and DNT, 682 nM)	CLO, IMD, TMX and DNT detection	(Johnson et al. 2021)
	Magnetic NPs	0.30 ng µL	*Phytophthora palmivora*	(Franco et al. 2019)
Localized surface plasmon resonance	AuNPs	5 ng/µL	TYLCV	(Razmi et al. 2019)
Physiological Stress Detection				
Electrochemical	AuNPs	18.5 nmol/L and 25.6 nmol/L for Cd and Pb, respectively	VN detection existing on the surface of plant cells during heavy metals (Cd or Pb) stress	(Wang et al. 2019b)
	Au nanostructures, PtNPs reduced GO nanocomposite films	43 pg/mL	IAA determination during salt stress	(Li et al. 2019)
Optical	AuNPs	2 fM	miRNAs concentration recognition serving as indicator in drought stress	(Vakilian 2019)
Localized surface plasmon resonance	AuNPs	0.33 µM	ABA detection	(Wang et al. 2017)
Toxic Chemical Detection				
Colorimetric	AuNPs	17.8 pg/mL	Ochratoxin A detection	(Liang et al. 2018b)
	AuNPs	10 ppb	Chlorpyrifos detection	(Mane et al. 2020)
	AuNPs	0.53 U/L	Omethoate detection	(Zhang et al. 2020a)
	AuNPs	0.04 mg/kg	Cyromazine detection	(Liu et al. 2020a)
	AuNPs	0.0197 µg/ml; 0.0186 µg/ml	λ-cyhalothrin detection	(Yang et al. 2021b)
	AuNPs	4.13 nM	Tebuconazole detection	(Xie et al. 2021)
Colorimetric/ electrochemical dual channel detection method	AuNPs	0.43 pg/mL and 35 pg/mL for electrochemical and colorimetric channel, respectively	Aflatoxin B1 detection	(Qian et al. 2020)
Electrochemical	AuNPs	0.086 µM	Acetamiprid detection	(Rapini et al. 2016)
	AuNPs	0.0169 nM	Diazinon detection	(Hassani et al. 2018)
	AuNPs	5.5 × 10^−14^ mol/L	Aflatoxin B1 detection	(Feng et al. 2020)
	AuNPs	22 pmol/L	Imidacloprid detection	(Pérez-Fernández et al. 2020)
	AuNPs	10 fM	CPS detection	(Talan et al. 2018)
	AuNPs	3.65 pM	Bisphenol A detection	(Nodehi et al. 2021)
	AuNPs	3.2 ng/mL	Deoxynivalenol (mycotoxin) detection	(Subak et al. 2021)
	Carbon nanotubes	6.3 pg/mL	CPS detection	(Sun et al. 2015)
	GO	0.033 ng/mL	CPS detection	(Jiao et al. 2017)
	Graphene nanosheets	0.002 ng/mL	Aflatoxin B1 detection	(Lin and Shen 2020)
	Gold-graphene quantum dot nanohybrid	3.33 × 10^−15^ M and 1.67 × 10^−14^ М for acetamiprid and omethoate, respectively	Acetamiprid and omethoate detection	(Ruiyi et al. 2022)
	Pt NPs	10 pM and 1 pM for atrazine and acetamiprid, respectively	Atrazine and acetamiprid detection	(Madianos et al. 2018)
	MWCNTs/DCHP	0.05 µg/L	CPS detection	(Chen et al. 2017)
Fluorescence resonance energy transfer	AuNCs	0.34 pg/mL and 0.53 pg/mL, respectively	Aflatoxin B1 and zearalenone detection	(Khan et al. 2019a)
Lateral flow assay	AuNPs	2 µg/kg and 1000 µg/kg, respectively	Aflatoxin B1 and fumonisins detection	(Di Nardo et al. 2017)
	AuNPs	3.44, 3.98 and 12.49 ng/mL, respectively	Parathion, parathion-methyl, and fenitrothion detection	(Zou et al. 2019)
	AuNPs	7.4 pg/mL	Zearalenone detection	(Xu et al. 2019)
	AuNPs	250 ng/mL	Triazophos detection	(Ge et al. 2020)
	Core-shell UCNPs	3 ng/mL	Ochratoxin A detection	(Jin et al. 2018)
	Europium Nanosphere	In maize: 0.16, 0.52 and 1.21 µg/kg, respectively while in peanut: 0.18, 0.57 and 1.47 µg/kg, respectively	Aflatoxin B1, zearalenone, and chlorothalonil detection	(Wang et al. 2019a)
Photoelectrochemical	AuNPs	0.05 pM	Acetamiprid detection	(Zheng et al. 2022a)
Surface enhanced Raman scattering	AgNPs	1 fM	Methyl parathion and thiram detection	(Zhu et al. 2020)
	Ag colloid	10^−9^ mol/L	Chlorpyrifos detection	(Ma et al. 2020)
	AuNPs	1 µM	CPS detection	(Xu et al. 2017)
	AuNPs	6, 60 and 600 ng/cm, respectively	Thiram tricyclazole and carbaryl detection	(Kwon et al. 2019)
Heavy Metal Detection				
Colorimetric	AuNPs	4.23 µM	Pd detection	(Anwar et al. 2018)
Aptasensors	SiO_2_ and graphene	0.0000001–0.01 pg/mL	Arsenic detection	(Uda et al. 2021)
Electrochemical	BiNP	0.8 and 0.5 µg/L for Pb (II) and Cd (II), respectively	Pb and Cd detection	(Zhao et al. 2022a)
	Graphene	28.2 ± 25.0 µM and 20.6 ± 14.8 µM for ammonium and nitrate, respectively	N detection in the soil, i.e., NH_4_+ and NO_3_− ions	(Garland et al. 2018)
	Graphene	0.17 µg/L	Pb detection	(Lu et al. 2019)
	Graphene	50 ppb	Pb detection	(Getachew et al. 2020)
	SWCNTs	3 fM	Hg detection	(Shi et al. 2017)
Surface-enhanced Raman spectroscopy	AuNPs	8 µg/kg	Cd detection	(Zuo et al. 2018)
Lateral flow assay Core-shell	AuNPs	0.18 ng/mL	Cd detection	(Xiao et al. 2018)
	AuNPs	2.53 nM	Hg detection	(Guo et al. 2020)
	UCNPs	5 ppb	Hg detection	(Jin et al. 2018)
Photoelectrochemical	Metal sulfide-graphene	2 nM	Cu detection	(Ge et al. 2019)

Abscisic acid, ABA; Ammonium ion, NH^4+^; Bismuth nanoparticle, BiNP; Cadmium, Cd; Carbon nanoparticles, CNPs; Chlorpyrifos, CPS; Citrus Tristeza Virus, CTV; Clothianidin, CLO; Copper, Cu; Dicyclohexyl phthalate, DCHP; Dinotefuran, DNT; Graphene oxide, GO; Gold, Au; Gold nanoclusters, AuNCs; Gold nanoparticles, AuNPs; Gold nanorods, AuNRs; Groundnut bud necrosis orthotospovirus, GBNV; Huanglongbing, HLB; Indole acetic acid, IAA; Imidacloprid, IMD; Lead, Pb; Mercury, Hg; micro Ribonucleic acids, miRNAs; Multi-walled carbon nanotubes, MWCNTs; Nanoparticles, NPs; Nitrate ion, NO^3−^; Nitrogen, N; Palladium, Pd; Platinum nanoparticles, PtNPs; Potato leaf roll virus, PLRV; Reduced graphene oxide, rGO; Silicon dioxide, SiO_2_; Silver, Ag; Silver nanoparticles, AgNPs; Single walled carbon nanotubes, SWCNTs; Thiamethoxam, TMX; Tomato Yellow Leaf Curl Virus, TYLCV; Upconverting nanoparticles, UCNPs; Vitronectin-like proteins, VN

Ultimately, nano biosensors offer effective and economical way for contaminant detection, environment friendly farming techniques and solving agricultural issues like ensuring food safety and overfertilizer use. In order to encourage sustainable agriculture and food security, the future prospects might include expanding the growth of nano biosensors to identify a wider variety of analytes, improving techniques involved in detection of disease and pathogens, as well as optimization of biosensing devices.

## Socio-economical, environmental, and health implications of nanoagriculture

A rapid increase in the use of different NPs in agriculture and food industry has raised serious concerns among scientists working to safeguard the environment by releasing a significant number of NPs into the environment. Nanoagriculture has both positive and negative impacts (Khan et al. 2019b; Pokharel et al. 2024).

The agri-food-nutrition and health sector has witnessed significant advancements, driven by the integration of newer technologies. As highlighted in Kumar et al. (2018), the strategic intervention of these technologies has led to the development of innovative value-added products. This trend has been particularly evident in the realm of nanotechnology, where metal-based NPs have demonstrated their potential to influence gene expression and regulate essential nutrient homeostasis, as explored by Chandra et al. (2021).

Although nanotechnology has great potential to advance food processing and create novel goods, it still faces significant challenges (Jain et al. 2018). It is important to consider the possible risks to human health and long-term environmental effects of the deliberate release of nanomaterials (Gilbertson et al. 2020; Pokharel et al. 2024). Nanotechnology faces many challenges, especially in terms of NP behavior. The NPs may be added directly, indirectly, or become isolated due to their movement. When compared to bulk materials, the properties of the material will be completely different (Kah and Kookana 2020). Therefore, it is crucial to comprehend the functions and toxicity of NPs. The NPs penetrate the biological barrier and penetrate cells and organs. Different chemical processes are utilized to manufacture the NPs, which results in significant environmental contamination and dangerous by-products (Chugh and Dar 2021).

Due to the incorporation of NPs into the food from the inadequate packaging, nanopackaged foods could pose a health risk. The type of packing matrix, degree of migration, rate of food ingestion, and toxicity of the nanomaterial all have a role in this impact (Kannan et al. 2020). Evidence of the health risks posed by NPs is becoming more prevalent day by day. The bioaccumulation, excessive intake, and increased activity of nano-based products have a negative impact on safety risks and health-related problems (Raj et al. 2021). The NPs occurring in packaging materials could enter food and accumulate in organs like stomach, kidney, liver, and small intestine (Fajardo et al. 2022).

Agriculture is administered by laws to ensure the security of food and feed sources (Hofmann et al. 2020). The use of smart nanomaterials in agriculture, a recently emerging technology, is constrained by the absence of adequate risk assessment and legislation to address safety issues (Lowry et al. 2019). When new technology is introduced, legislators often face hurdles, especially when the technology’s commercially represented benefits raise concerns about environmental, and human health risks (Gottardo et al. 2021).

Beyond these immediate concerns, nanotechnology and genomics in agriculture also pose socio-economic, environmental, and biosafety challenges that must be carefully managed to ensure equitable and sustainable development.

Socio-economically, these innovations may widen the gap between large agribusinesses and smallholder farmers, as the high costs of adopting nanotechnology and genomics can limit access for farmers in developing countries. Additionally, the rise of proprietary seeds and nanotechnologies controlled by large corporations can lead to dependency, reducing the autonomy of farmers and exacerbating inequalities in rural areas. Environmentally, nanomaterials can persist in ecosystems, with potential effects on soil, water, and biodiversity that are not yet fully understood. Genomic innovations, such as genetically modified crops, may further disrupt ecosystems by affecting non-target species and reducing genetic diversity, particularly if transgenes spread to wild relatives.

Biosafety concerns also arise from the use of nanotechnology and genomics in food production, as NPs could accumulate in the food chain and unintended genetic modifications in crops could pose health risks. Ensuring food safety and environmental sustainability will require robust regulatory frameworks, especially in developing countries where the necessary infrastructure for monitoring and managing these risks may be lacking. Ethical considerations, including public perceptions of genome editing and the potential for irreversible ecological impacts, add another layer of complexity to the adoption of these technologies.

## Future aspects of nanotechnology in agriculture

Nanotechnology is playing a crucial role in enhancing crop production in the agricultural sector. More efforts are required to study the toxic effects of NPs on soil properties, soil fertility, and plant growth and development. Plant growth and soil-plant system may be affected by the presence of NPs in the soil. The safety, interaction with plants, side effects, and possible mechanism of action need some more research in this area.

The nano agrochemicals, practices such as nano fertilizers and nano pesticides, are well recognized to efficiently provide crop protection from pests, enhance plant growth and nutrition, and increase agricultural output. New nanomaterials and nanotechnology are becoming popular in the agricultural sector in which research has focused on nano formulated pesticide development (Jampílek and Kráľová 2019). Several nano pesticide patents have been developed by many agricultural companies including Badische Anilin und Soda Fabrik (BASF), Monsanto, Bayer, Syngenta, and Dow AgroScience (Peters et al. 2016; Kah et al. 2019). It’s also important to evaluate the bio effectiveness and long-term toxicity of a substance to non-target organisms.

The physicochemical characterization of nanomaterials must be emphasized, as well as how the quality and traits influence the environment (Tomak et al. 2021). In agriculture sector, there are no regulatory laws for nanotechnology field. That’s why for their safe implementation, effective laws and regulations are needed. Nanomaterials only address the broader concepts, and the actual law is in its infancy due to these limitations.

To fulfill the demands of the future, the food and agricultural industries must switch to green technologies. Agro nanotechnology is essential for sustainable farming as well as fulfilling the needs of future generations. We must concentrate on synthesis, screening, and nanomaterial optimization of green technology for different kinds of plants prior to commercialization. Nanomaterials’ properties and stability can be modified to alter their efficacy and behaviour.

Nanotechnology has the power to revolutionize agriculture. Future research should focus on exploring the long-term impacts of nanomaterials on ecosystems and human health. Additionally, there is a need to develop standardized protocols for the safe and sustainable application of nanotechnology in agricultural practices (Saritha et al. 2022). By addressing these challenges and capitalizing on the opportunities presented by emerging technologies, we can create a more resilient, efficient, and sustainable agri-food-nutrition and health sector.

## Conclusion

This review critically explored the integration of nanotechnology and genetic innovations in agriculture, highlighting the strides made, persistent challenges, and potential opportunities for future advancements. This fusion has showcased promising solutions for improving agricultural practices. Use of nanomaterials for targeted delivery in agriculture has shown potential in optimizing resource utilization and minimizing environmental impact. Simultaneously, genetic innovations, especially in precision gene editing like CRISPR, have shown opportunities to tailor crop modifications for better yields, resilience, and nutritional value. However, several critical issues persist, demanding comprehensive solutions for effective genome delivery, modern hybrid nanomaterial design, and enhancement of techniques such as pollen magnetofection and CRISPR strategies. Moreover, the impact of nanomaterials on plant and soil health, alongside their potential toxicity, remains a critical concern. In addition, regulatory framework guiding the usage of nanomaterials in agriculture is still evolving, highlighting comprehensive guidelines for safe implementation.

Further research into nanomaterial properties, their stability, and optimization techniques prior to commercialization could pave the way for more effective and safer applications in farming. Additionally, refining delivery mechanisms for genetic modifications and advancing precision editing technologies will play pivotal roles in improving agriculture practices.

In conclusion, the fusion of nanotechnology and genetic innovations represents a transformative approach that can revolutionize farming practices, ensuring sustainable and efficient food production for generations to come.

## Data Availability

No datasets were generated or analysed during the current study.
